# Sustained release of a GLP-1 and FGF21 dual agonist from an injectable depot protects mice from obesity and hyperglycemia

**DOI:** 10.1126/sciadv.aaz9890

**Published:** 2020-08-26

**Authors:** C. A. Gilroy, M. E. Capozzi, A. K. Varanko, J. Tong, D. A. D'Alessio, J. E. Campbell, A. Chilkoti

**Affiliations:** 1 Department of Biomedical Engineering, Duke University, Durham, NC 27708, USA.; 2 Department of Nutritional Sciences and Toxicology, University of California, Berkeley, CA 94720, USA.; 3 Duke Molecular Physiology Institute, Duke University, Durham, NC 27701, USA.; 4 Department of Medicine, Duke University, Durham, NC 27701, USA.; 5 Department of Pharmacology and Cancer Biology, Duke University, Durham, NC 27701, USA.

## Abstract

There is great interest in identifying a glucagon-like peptide-1 (GLP-1)–based combination therapy that will more effectively promote weight loss in patients with type 2 diabetes. Fibroblast growth factor 21 (FGF21) is a compelling yet previously unexplored drug candidate to combine with GLP-1 due to its thermogenic and insulin-sensitizing effects. Here, we describe the development of a biologic that fuses GLP-1 to FGF21 with an elastin-like polypeptide linker that acts as a sustained release module with zero-order drug release. We show that once-weekly dual-agonist treatment of diabetic mice results in potent weight-reducing effects and enhanced glycemic control that are not observed with either agonist alone. Furthermore, the dual-agonist formulation has superior efficacy compared to a GLP-1/FGF21 mixture, demonstrating the utility of combining two structurally distinct peptides into one multifunctional molecule. We anticipate that these results will spur further investigation into GLP-1/FGF21 multiagonism for the treatment of metabolic disease.

## Teaser

A type 2 diabetes therapeutic merges two complementary drugs with a depot-forming linker to enable once-weekly dosing.

## INTRODUCTION

More than 34 million people in the United States have diabetes, with the number of diagnosed individuals growing rapidly, and obesity serving as a major risk factor (*1*). Type 2 diabetes (T2D) accounts for most cases and is characterized by a state of insulin resistance and impaired ability to maintain glucose homeostasis. Treatment beyond lifestyle changes generally begins with oral antidiabetic agents, but these medicines only have transitory benefit, and the progressive nature of T2D requires therapeutic intensification to include insulin within 5 to 10 years for many patients (*2*–*4*). Moreover, current treatments, including insulin, are frequently accompanied by weight gain. Thus, there is a pressing need for the development of drugs or drug combinations that maximize glycemic control while promoting weight loss (*5*).

Glucagon-like peptide-1 (GLP-1) receptor agonists (GLP-1RAs) are a class of incretin peptides that enhances glucose-stimulated insulin secretion and reduces food intake (*6*). This class of injectable drugs decreases fasting blood glucose levels and improves long-term glycemic control while inducing mild to moderate weight loss in most patients (*7*). The advent of GLP-1RAs has added a notable dimension to T2D management, allowing a greater number of patients to reach their glycemic targets than was previously achieved with conventional medicines (*7*), and these agents are now recommended before insulin for treatment of T2D (*8*). However, despite their unique mechanism of action and solid clinical track record, even GLP-1RAs are not entirely sufficient for chronic glucose control in patients with T2D, and the weight loss induced by these drugs rarely corrects obesity. Moreover, higher doses capable of augmenting weight loss are often limited by gastrointestinal side effects (*7*).

GLP-1–based multiagonism is a therapeutic strategy that has gained substantial interest recently, with the goal of promoting weight loss and improving glycemic control beyond that afforded by GLP-1RAs alone. Peptides with balanced coagonism for GLP-1 and glucose-dependent insulinotropic polypeptide (GIP) have been shown to enhance weight loss and glycemic control, and weight loss is further amplified when the thermogenic action of glucagon agonism is incorporated into the multiagonist peptide (*9*). Weight reduction associated with GLP-1/GIP/glucagon multiagonism is believed to be, in part, a consequence of increased fibroblast growth factor 21 (FGF21) plasma levels induced by glucagon signaling (*10*). While glucagon agonism remains controversial because of its glycemia-elevating effects through hepatic glucose production activation (*11*), we propose that FGF21 might serve as a suitable candidate for a GLP-1 pairing.

FGF21 was recently identified as an important metabolic hormone (*12*), targeting the liver, pancreas, and adipose tissues to regulate insulin sensitivity, energy expenditure, and lipid metabolism (*13*). FGF21 analogs are being pursued as drug candidates for treating T2D as well as obesity and nonalcoholic steatohepatitis, and emerging human clinical trial data appears promising (*14*–*16*). We hypothesized that fusing FGF21 with GLP-1 would provide the thermogenic benefits to complement reduced food intake for maximal weight reduction, without diminishing the glucose control afforded by GLP-1. Furthermore, rather than localizing glycemic effects to the pancreas—as is the case with members of the glucagon superfamily—the FGF21 component has the potential to further improve glucose control by acting on peripheral tissues to increase insulin sensitivity (*13*).

To this end, we sought to design a drug capable of both GLP-1 and FGF21 receptor agonism and report here the development of a simple yet innovative strategy that incorporates an elastin-like polypeptide (ELP) to act as both a flexible linker between GLP-1 and FGF21 and as a sustained release module. Our dual-agonist drug robustly improves metabolic parameters, restoring glycemic control and inhibiting weight gain in a mouse model of obesity and hyperglycemia. Meanwhile, the ELP addresses one of the major hurdles in developing GLP-1 and FGF21 as drugs—their short plasma half-lives (minutes and hours, respectively)—by reducing the frequency of required administration to once weekly. These data provide insight into the unexpectedly potent combined effects of GLP-1 and FGF21 while establishing a method for incorporating structurally distinct peptide-based drugs into a multiagonist format.

## RESULTS

### GLP1-ELP and ELP-FGF21 cotreatment has potent weight-reducing effects

To test our hypothesis that GLP-1 and FGF21 act additively—or better—to control glycemia and inhibit weight gain, we carried out a short-term pilot study comparing a GLP-1/FGF21 combination therapy to each respective single-drug treatment. This pilot study used long-acting analogs of GLP-1 and FGF21, each fused to an ELP, that we previously developed and validated for the treatment of T2D (*17*, *18*). ELPs are repetitive peptide polymers characterized by a (VPGX_aa_G)*_n_* sequence, where “X_aa_” is any amino acid besides proline and “*n*” is the number of repeats (*19*, *20*). A notable feature of ELPs is their reversible lower critical solution temperature (LCST) phase behavior in aqueous medium (*21*). ELPs have a distinct and tunable “transition temperature” (*T*_t_)—also referred to as the cloud point temperature. Below their *T*_t_, ELPs are miscible in water, and above their *T*_t_, they form a water-immiscible coacervate (*21*), and this thermal responsiveness is retained when an ELP is genetically fused to a peptide or protein drug (*22*). By manipulating the *T*_t_—via choice of the X_aa_ residue and the molecular weight (*23*)—an ELP-drug fusion can be designed to form a depot under the skin that steadily releases molecules into systemic circulation (*17*, *18*, *24*). GLP1-ELP and ELP-FGF21 fusions are active in vitro and form subcutaneous depots capable of blood glucose–lowering effects for at least 5 days in diabetic mice following a single injection (*17*, *18*).

**Fig. 1:**
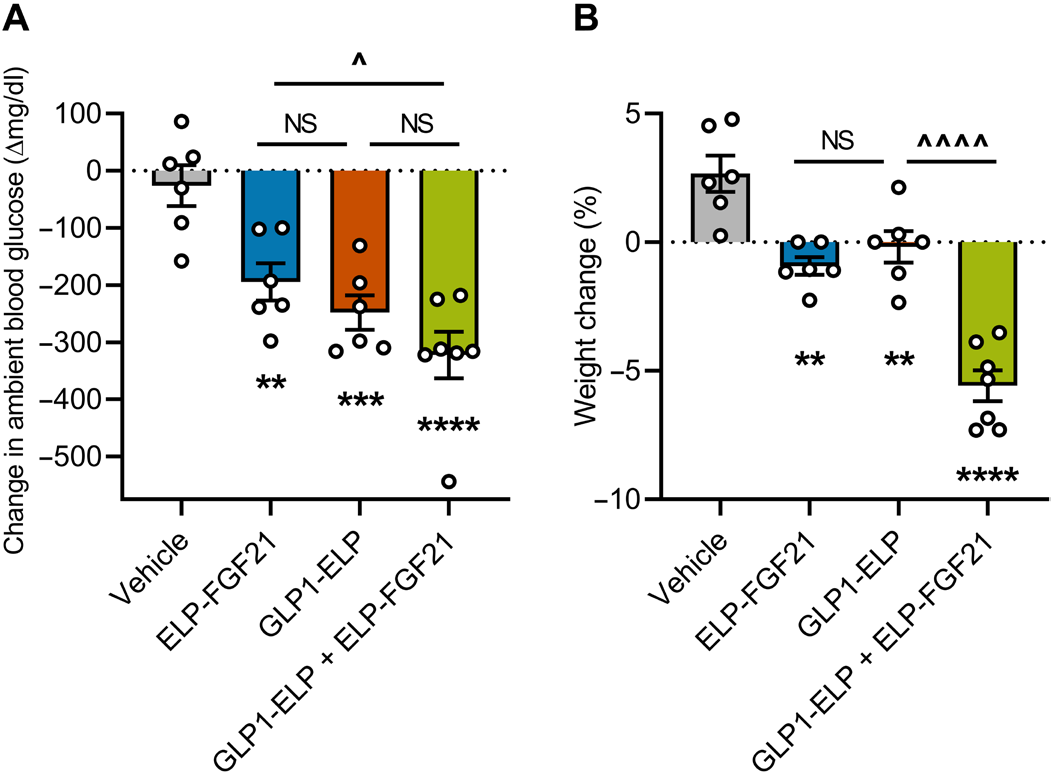
GLP-1 and FGF21 cotreatment augments the blood glucose–lowering and weight gain–inhibiting effects of single-drug treatment. Six-week-old *db/db* mice (*n* = 6 to 7) were subcutaneously injected with ELP-FGF21 (1000 nmol/kg), GLP1-ELP (1000 nmol/kg), or GLP1-ELP and ELP-FGF21 (1000 nmol/kg each). Ambient blood glucose levels (**A**) and body weights (**B**) were measured 48 hours after injection and reported as a magnitude change from pretreatment baseline and a percentage change from preinjection weight. Data are presented as means ± SEM and were analyzed by one-way ANOVA, followed by Tukey’s tests. *, treatment compared to vehicle; ^, comparisons between treatments; ^*P* < 005, **P < 0.01, ***P < 0.001, and ****/^^^^*P* < 0.0001. NS, not significant (*P* > 0.05).

*Db/db* mice were injected subcutaneously with GLP1-ELP, ELP-FGF21, an equimolar mixture of GLP1-ELP and ELP-FGF21, or vehicle control. Ambient blood glucose levels and body weights were measured 48 hours after injection and reported as a change from preinjection baseline. All treatments significantly reduced blood glucose levels compared to vehicle, while combination treatment resulted in blood glucose levels that trended even lower than each respective single drug (Fig. 1A). Treatment with ELP-FGF21 or GLP1-ELP effectively inhibited weight gain (−0. 9 ± 0.3 and −0.2 ± 0.6%) compared to vehicle-treated mice, which gained 2.7 ± 0.7% body weight in 48 hours (Fig. 1B). In contrast, mice treated with the combination of ELP-FGF21 and GLP1-ELP exhibited a robust 5.6 ± 0.6% reduction in body weight (Fig. 1B). Together, these data suggest that GLP-1 and FGF21 act at least additively to induce weight loss and possibly to improve glycemic control in diabetic mice, motivating the development of a modular drug with GLP-1 and FGF21 dual agonism while retaining the ELP component for sustained release.

### Design and production of a unimolecular GLP-1/FGF21 dual agonist

The dual agonist was designed as a head-to-tail polypeptide fusion protein, with GLP-1 located at the N terminus, FGF21 at the C terminus, and an intervening ELP (“GLP1-ELP-FGF21”). This orientation provided a solvent-exposed N terminus for GLP-1, and an exposed C terminus for FGF21, both of which are essential to activate their respective receptors (*6*, *25*), while the linear architecture enabled facile synthesis and scale-up in a bacterial expression system. The ELP served a dual role as both a flexible linker—providing sufficient physical separation between the two drugs to enable dual receptor binding—and a module to create an injectable depot and thereby enable sustained release of the drug from the injection site.

**Fig. 2:**
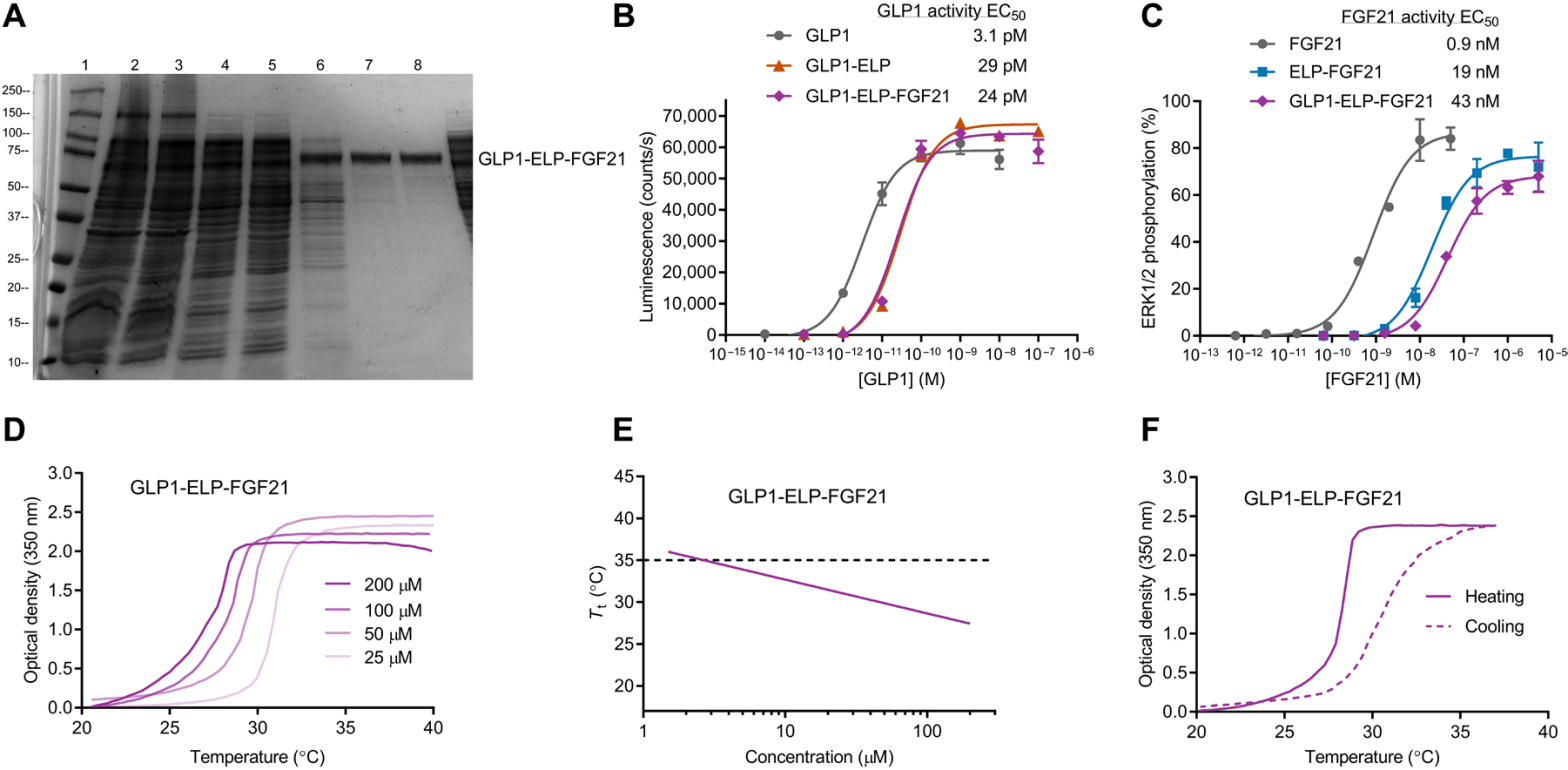
A recombinant GLP1-ELP-FGF21 fusion protein has dual agonism and LCST phase behavior. (**A**) SDS-PAGE analysis of the 72-kDa fusion protein following recombinant expression in *E. coli* and ITC-based purification. 1, molecular weight ladder (kilodaltons); 2, cell lysate; 3, insoluble lysate fraction; 4, soluble lysate fraction; 5, hot spin supernatant; 6 to 8, ITC rounds 1 to 3. (**B** and **C**) In vitro GLP-1 and FGF21 activity assays for GLP1-ELP-FGF21. GLP-1R agonism (B) was measured by quantifying adenosine 3′,5′-monophosphate (cAMP) production in human embryonic kidney (HEK) 293 cells stably expressing the GLP-1R and the cAMP-inducible luciferase reporter. Cells were stimulated for 5 hours with GLP1-ELP-FGF21, the GLP1-ELP single-agonist control, or native GLP-1. FGF21 receptor agonism (C) was measured by quantifying extracellular signal–regulated kinase 1/2 (ERK1/2) phosphorylation in HEK293 cells stably expressing FGF receptor 1 (FGFR1) and β-klotho and by normalizing phospho-ERK1/2 to total ERK1/2. Cells were stimulated for 5 min with GLP1-ELP-FGF21, the ELP-FGF21 single-agonist control, or native FGF21. Data are presented as means ± SEM, *n* = 3. (**D** to **F**) LCST phase transition behavior of GLP1-ELP-FGF21. (D) The optical density (OD) at 350 nm of GLP1-ELP-FGF21 at the indicated concentration in phosphate-buffered saline (PBS) was measured as a function of temperature, with temperature ramped at a rate of 1°C/min. (E) Turbidity versus temperature scans were repeated as in (D) for the indicated concentrations (*n* = 3). *T*_t_ values were defined as the temperature corresponding to the 50% maximum OD and are plotted as a function of fusion protein concentration. The horizontal dashed line indicates the approximate temperature of the subcutaneous space in a mouse (*27*). (F) A turbidity scan was repeated for GLP1-ELP-FGF21 at an injection-relevant concentration (150 μM), with the temperature ramped up to 37°C and then down to 20°C.

GLP1-ELP-FGF21 used mutations in FGF21 to promote protein stability (*18*) and in GLP-1 to stabilize the α helix and protect the N terminus from proteolytic cleavage (*24*). GLP-1 also incorporated a di-alanine (AA) leader that facilitated recombinant expression, but that is removed in vivo by dipeptidyl peptidase 4 (DPP4) to expose an active N terminus (*24*). The ELP sequence was strategically chosen on the basis of its *T*_t_, and it consisted of (VPGXG)_120_ with a 4:1 valine:alanine ratio in the X_aa_ residue position (see table S1 for the complete amino acid sequence). The GLP1-ELP-FGF21 fusion protein was recombinantly expressed in *Escherichia coli* and was purified from crude cell lysate by inverse transition cycling (ITC), a nonchromatographic method developed by our research group that exploits that LCST phase transition behavior imparted to the ELP fusion by the ELP tag (*22*, *26*). A 72-kDa band associated with the full-length GLP1-ELP-FGF21 fusion product was visible in SDS–polyacrylamide gel electrophoresis (PAGE) throughout the purification process (Fig. 2A), and ITC purification alone was sufficient to isolate the dual agonist from contaminants.

### GLP1-ELP-FGF21 has dual agonism at the GLP-1 and FGF21 receptors

The half-maximal effective concentration (EC_50_) for the GLP-1 and FGF21 dual-agonist components were measured using in vitro activity assays in cells stably expressing either the GLP-1R or the FGF21 receptor complex. Fusion of GLP-1 to an ELP increased the GLP-1R EC_50_ approximately 10-fold (Fig. 2B), which agrees with our previous findings (*17*), while the GLP-1R EC_50_ of GLP1-ELP-FGF21 (23.9 ± 5.7 pM) was not different from that of GLP1-ELP (29.5 ± 5.0 pM) (Fig. 2B). Fusion of FGF21 to an ELP increased the FGF21 receptor EC_50_ approximately 20-fold (Fig. 2C), which is also consistent with our previous reports (*18*). The dual agonist had a marginally greater FGF21 receptor EC_50_ (43.2 ± 8.4 nM) compared to ELP-FGF21 (18.8 ± 4.5 nM) (Fig. 2C), but this difference was not statistically significant (*P* > 0.05). Together, these data demonstrate that simultaneous presentation of GLP-1 and FGF21 on an ELP does not significantly affect the activity of each drug.

### Phase transition behavior of GLP1-ELP-FGF21 is suitable for depot formation

The LCST phase transition behavior of the GLP1-ELP-FGF21 fusion was evaluated by monitoring the optical density (OD) of a solution of the fusion protein as a function of temperature, defining the *T*_t_ as the temperature at which the solution becomes turbid. On the basis of previous optimization studies by our group (*17*, *18*), we have identified a target *T*_t_ range between 27° and 32°C as suitable for depot formation—triggered by body heat—with ELP-drug release kinetics that are appropriate for once-weekly dosing. An ELP fusion with a *T*_t_ of <27°C forms an excessively stable coacervate that exhibits poor drug absorption, while a fusion with a *T*_t_ near 35°C [the temperature of the mouse subcutaneous space (*27*)] exhibits a bolus-like release profile (*17*). GLP1-ELP-FGF21 was confirmed to have LCST phase change behavior, with a *T*_t_ between 27° and 29°C at the injection-relevant concentration range of 100 to 200 μM (Fig. 2D). The *T*_t_ was concentration dependent (Fig. 2E), and the dual-agonist phase change behavior was reversible (Fig. 2F). The reversibility and inverse dependence of *T*_t_ on fusion protein concentration (*28*) are attributes critical to the controlled release capabilities of ELP-based drug depots: As fusion protein molecules at the depot margin are diluted, their *T*_t_ rises above body temperature, thereby reversing the LCST phase transition and allowing the release of ELP-drug fusion molecules from the depot.

### A GLP1-ELP-FGF21 dual-agonist fusion protein has sustained dose-dependent effects on body weight and glycemia

**Fig. 3:**
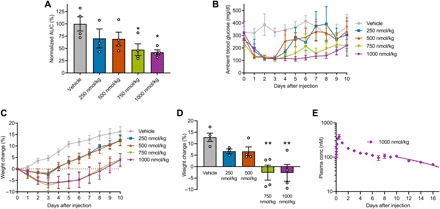
The GLP1-ELP-FGF21 dual-agonist fusion protein has potent and sustained effects on glycemia and body weight. (**A** to **D**) Six-week-old *db/db* mice (*n* = 3 to 4) received a single subcutaneous injection of GLP1-ELP-FGF21 at the indicated dose or vehicle. Ambient blood glucose levels were measured every 24 hours until animals returned to baseline levels and are reported as blood glucose versus time AUC or raw values (A and B). Body weights were recorded daily and are reported as a percentage change from preinjection weight over time (C) or on day 7 after injection (D). (**E**) GLP1-ELP-FGF21 (1000 nmol/kg) was subcutaneously administered to 6-week-old *db/db* mice (*n* = 4) as a radiolabeled protein, blood samples were collected at indicated time points following injection, and plasma gamma counts were correlated to fusion protein concentration. Regression curves were fit to the terminal portion of the dataset, and data could be described by either a first-order (dotted) or a zero-order (solid) elimination model. Data are presented as means ± SEM and were analyzed by one-way ANOVA, followed by Dunnett’s tests. *P < 0.05 and **P < 0.01.

The dual-agonist drug was next tested for efficacy in diabetic mice. The *db/db* mouse model was selected because of its extreme degree of hyperglycemia paired with obesity, as high baseline body weight and glycemic levels provided a large window to identify additive effects of dual agonism. Mice were administered either a single subcutaneous injection of GLP1-ELP-FGF21 at the indicated dose or vehicle. Ambient blood glucose levels were measured daily until all cohorts returned to baseline levels. Significant reductions in blood glucose versus time area under the curve (AUC) were observed at the two highest doses tested (750 and 1000 nmol/kg) (Fig. 3A); however, raw blood glucose versus time data revealed that it was not effect size but rather effect duration that increased in a dose-dependent manner (Fig. 3B). All doses reduced blood glucose levels from >300 to <150 mg/dl (Fig. 3B), with effects persisting for 4 days at the lowest dose (250 nmol/kg) and 8 days at the highest dose tested (1000 nmol/kg) (Fig. 3B). This dose-response trend aligns with previous observations (*17*) and is consistent with an ELP-based depot platform that releases drug at a steady rate for a duration proportional to initial dosing size. Compared to vehicle control, all tested doses of GLP1-ELP-FGF21 had an inhibitory effect on weight gain, while the two highest doses tested induced weight loss—reducing body weights by up to 7.2 ± 2.3% (Fig. 3C). On day 7 after treatment, a net weight loss effect persisted in the groups (750 and 1000 nmol/kg) (−2.6 ± 3.3 and −2.7 ± 3.7%, respectively) (Fig. 3D).

To confirm that sustained efficacy was the result of prolonged drug in circulation—as is observed when an ELP depot forms at the site of injection (*17*, *18*)—plasma drug levels were measured over time following a single subcutaneous injection of GLP1-ELP-FGF21 to *db/db* mice. Aside from a modest burst release in the first 24 hours, plasma drug levels remained steady near 100 nM out to day 10 (Fig. 3E), at which point drug levels decreased at a rate consistent with first-order elimination (*29*). When a linear regression curve was fit to the terminal portion of the data, the absorption half-life was calculated to be 7.6 ± 1.1 days (table S2). The data were described nearly as well by a zero-order elimination model (*R*^2^ = 0.81) as by a first-order model (*R*^2^ = 0.85) (Fig. 3E and table S2). The likely zero-order release kinetics observed here are consistent with ELP-based drug depots observed previously (*17*) and correspond to the release of fusion molecules into circulation at a constant rate. When pharmacokinetic data were analyzed alongside the blood glucose–versus–time efficacy data (1000 nmol/kg) (Fig. 3B), 100 nM appeared to be the minimal therapeutic concentration, as blood glucose levels returned to baseline in the same time frame that serum drug levels dropped below 100 nM (on or after day 10).

In summary, treatment of obese and hyperglycemic mice with a GLP-1/FGF21 dual-agonist drug had potent and sustained effects on body weights and ambient blood glucose levels. A single injection was sufficient to maintain therapeutic drug levels and protect mice from hyperglycemia and weight gain for >7 days, demonstrating the suitability of a GLP1-ELP-FGF21 depot for a once-weekly dosing scheme. A dose of 1000 nmol/kg was identified as yielding a maximal therapeutic effect over the intended dosing cycle and was selected for further evaluation.

### A GLP1-ELP-FGF21 dual agonist outperforms a long-acting GLP-1RA and a GLP-1/FGF21 drug mixture in diabetic mice

To elucidate the relative contribution of GLP-1 and FGF21 to the potent in vivo effects of the dual agonist, as well as understand the impact of incorporating both drugs into a single molecule, we next compared the efficacy of GLP1-ELP-FGF21 with that of a GLP1-ELP single-agonist monotherapy, an ELP-FGF21 monotherapy, and an equimolar mixture of GLP1-ELP and ELP-FGF21. To ensure consistency across treatments, the same ELP sequence was used in each drug formulation. The previously characterized ELP-FGF21 fusion (*18*) incorporates an ELP of equal length and composition to that comprising the dual agonist (table S1), however, a fusion of GLP-1 to the same ELP was not available from previous studies; hence, we produced the relevant GLP1-ELP fusion (table S1). To do so, a vector encoding GLP-1 fused at its C terminus to the ELP of interest [(VPGXG)_120_ with a 4:1 valine:alanine ratio at the X_aa_ residue position] was expressed in *E. coli* and purified by ITC as previously described (*17*). A 52-kDa band associated with the GLP1-ELP fusion was visible by SDS-PAGE following purification (fig. S1A). The appropriately designed GLP1-ELP fusion was tested for GLP-1R agonism and exhibited the anticipated 10-fold increase in EC_50_ compared to native GLP-1 (fig. S1B).

The LCST phase transition behavior was evaluated for GLP1-ELP and the equimolar mixture of GLP1-ELP and ELP-FGF21, revealing *T*_t_ values of 27.5°and 28°C, respectively, at the injection-relevant concentration of 200 μM (fig. S1, C and D). The *T*_t_ values were concentration dependent (fig. S1, E and F), and the phase behavior of each fusion/fusion mixture was reversible (fig. S1, G and H). Note that the LCST phase transition behavior of ELP-FGF21 has been characterized previously (*18*).

**Fig. 4:**
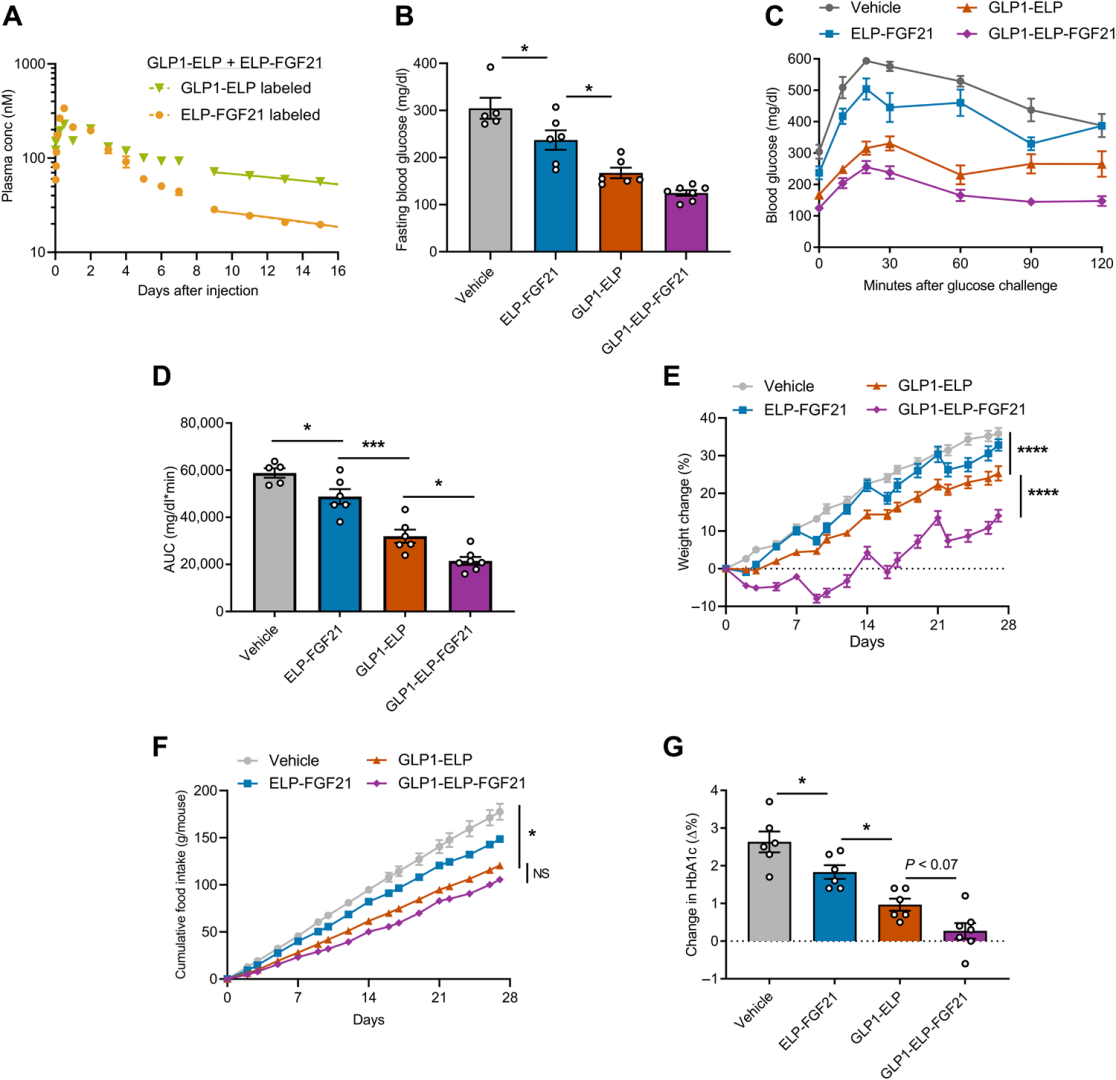
A GLP1-ELP-FGF21 dual agonist confers greater glycemic control and protection from weight gain compared to a long-acting GLP-1RA. (**A**) A mixed ELP depot releases GLP-1 and FGF21 at different rates. Six-week-old *db/db* mice (*n* = 4 to 5) received a single subcutaneous injection consisting of a 1:1 mixture of the synthesized GLP1-ELP and previously reported ELP-FGF21 (*18*). The mixture was tested once when GLP1-ELP was radiolabeled and once when ELP-FGF21 was radiolabeled. Fusions were injected at 200 μM and dosed at 1000 nmol/kg each of GLP1-ELP and ELP-FGF21. Blood samples were collected at indicated time points following injection, and plasma gamma counts were measured and correlated to fusion protein concentration. Lines represent regression curves fit to the terminal portion of each dataset. Data can be described by both the first-order (dotted) and zero-order (solid) elimination models. (**B** to **G**) Six-week-old *db/db* mice (*n* = 6 to 7) were treated weekly for 4 weeks with GLP1-ELP-FGF21, GLP1-ELP, ELP-FGF21, or PBS vehicle. Fusion proteins were administered subcutaneously at 1000 nmol/kg. (B to D) Glucose challenge. Seventy-two hours after the first treatment cycle, mice were fasted 5 hours, baseline blood glucose levels were measured (B), and mice were injected intraperitoneally with glucose (0.75 g/kg). Blood glucose levels were measured at indicated time points (C), and blood glucose versus time AUC values were calculated (D). (E and F) Body weights and food consumption were measured every 1 to 2 days and are reported as percentage change from preinjection weights (E) and cumulative food intake per mouse (F). (G) Before the first treatment (day 0) and 6 days following the final treatment (day 27), percent glycated hemoglobin A1c (%HbA1c) was measured in all cohorts and reported as a magnitude change from prestudy values. Data are presented as means ± SEM and were analyzed by one-way ANOVA or two-way repeated-measures ANOVA, followed by Dunnett’s tests. *P < 0.05, ***P < 0.001, and ****P < 0.0001.

Next, pharmacokinetic profiles were evaluated for the synthesized GLP1-ELP fusion, as well as the equimolar mixture of GLP1-ELP and ELP-FGF21. Both GLP1-ELP and the mixture exhibited steady plasma drug levels consistent with sustained release from a subcutaneous depot (Fig. 4A and fig. S1I). The GLP1-ELP monotherapy depot behaved similarly to that of the dual agonist, releasing fusion molecules into circulation at a rate that could be described by the zero-order elimination model (table S2) and resulting in plasma drug levels hovering near 100 nM for at least 10 days (fig. S1I). The 1:1 mixed depot released GLP1-ELP and ELP-FGF21 fusion molecules at different rates (Fig. 4A). The ELP-FGF21 component reached a higher maximum serum concentration (*C*_max_) than the GLP1-ELP component (table S2), and ELP-FGF21 plasma levels dropped below 100 nM by day 4, while GLP1-ELP plasma levels hovered steadily around 100 nM for 7 days (Fig. 4A). Although absorption of each component of the mixture fit the zero-order release model (table S2), neither GLP1-ELP nor ELP-FGF21 maintained steady plasma levels for as long as GLP1-ELP-FGF21 did, suggesting a pharmacokinetic advantage to delivering the two drugs in a unimolecular format. Note that sustained release of ELP-FGF21 from a subcutaneous depot has been previously reported (*18*).

After establishing appropriate controls, we compared the acute and chronic metabolic effects of dual-agonist treatment to that of the respective single-agonist ELP fusion proteins. *Db/db* mice were treated weekly for 4 weeks with GLP1-ELP-FGF21 (1000 nmol/kg), GLP1-ELP (1000 nmol/kg), ELP-FGF21 (1000 nmol/kg), or vehicle. An intraperitoneal glucose tolerance test (GTT) (0.75 mg/kg) was performed 3 days after the first dosing cycle, when body weights were consistent across treatment groups (fig. S2A) and each drug was at a maximally therapeutic plasma concentration (Fig. 3E and fig. S1I) (*18*). Fasting blood glucose levels measured before the GTT revealed a stepwise decrease following treatment with ELP-FGF21, GLP1-ELP, and GLP1-ELP-FGF21, respectively, with levels trending lowest in the dual-agonist cohort (125 ± 6.0 mg/dl) (Fig. 4B). All treatments improved glucose tolerance compared to vehicle (Fig. 4C) and were further analyzed by AUC (Fig. 4D), which sets a consistent baseline of 0 mg/dl due to the varying *t* = 0 blood glucose levels for each respective cohort. ELP-FGF21 monotherapy treatment significantly decreased GTT AUC compared to vehicle, while GLP1-ELP monotherapy significantly decreased AUC compared to ELP-FGF21 (Fig. 4D). Meanwhile, GLP1-ELP-FGF21 treatment resulted in the lowest AUC (Fig. 4D) and was the only cohort to recover to near-baseline levels within 60 min (Fig. 4C), indicating a degree of glucose tolerance afforded only when FGF21 and GLP-1 are administered in combination.

Chronic dosing with the GLP1-ELP monotherapy significantly inhibited weight gain over the 4-week treatment period compared to vehicle (Fig. 4E). The reduced rate of weight gain in this cohort was likely due in part to a significant reduction in food intake (Fig. 4F), which is consistent with the anorectic effect associated with GLP-1RA therapy. Dual-agonist treatment further reduced weight gain compared to GLP1-ELP monotherapy over the 4-week study (+14.1 ± 1.6% versus +25.3 ± 1.9%, respectively) (Fig. 4E), despite GLP1-ELP-FGF21–treated mice consuming chow at an equivalent rate to GLP1-ELP–treated mice (Fig. 4F). Thus, the weight reduction observed in the dual-agonist cohort could not be attributed exclusively to an anorectic effect and likely incorporated an additional mechanism of action involving energy expenditure. Note that ELP-FGF21 monotherapy did not induce a significant effect on body weight (Fig. 4E), indicating that cooperative action between FGF21 and GLP-1 is necessary for realizing the full weight loss effect.

Percent glycated hemoglobin A1c (%HbA1c) was measured before the initiation of treatment (day 0) and at the termination (day 27) of the chronic dosing study. %HbA1c reflects mean circulating glucose levels over time and only shifts with red blood cell turnover (*30*). The average life span for a red blood cell is ~40 days in mice (*31*), and therefore, a 4-week study was sufficient to elicit noticeable changes in %HbA1c. Both ELP-FGF21 and GLP1-ELP monotherapies significantly inhibited %HbA1c elevation relative to control, although chronic GLP1-ELP treatment was not sufficient to prevent a net of 1.0 ± 0.2% rise from days 0 to 27 (Fig. 4G and fig. S2B). The dual-agonist cohort exhibited the greatest degree of long-term glycemic control, with a minimal +0.3 ± 0.2% change in %HbA1c (*P* < 0.07 compared to GLP1-ELP) (Fig. 4G). Thus, treatment with the GLP1-ELP-FGF21 dual-agonist drug affords superior glycemic control compared to equimolar dosing of a long-acting GLP-1RA, likely through coordinated action of the GLP-1 and FGF21 components to enhance insulin secretion and increase insulin sensitivity, respectively.

Next, the metabolic effects of the GLP-1/FGF21 combination therapy were evaluated with respect to drug format. *Db/db* mice received a single subcutaneous injection of GLP1-ELP-FGF21 (1000 nmol/kg) or a mixture of each of GLP1-ELP (1000 nmol/kg) and ELP-FGF21 (1000 nmol/kg), and an ELP-only formulation (1000 nmol/kg) was included as a negative control. Body weights and ambient blood glucose levels were measured daily until the cohorts returned toward baseline, and an intraperitoneal GTT (0.75 mg/kg) was performed 6 days after treatment to evaluate how glycemic control was maintained over time.

**Fig. 5:**
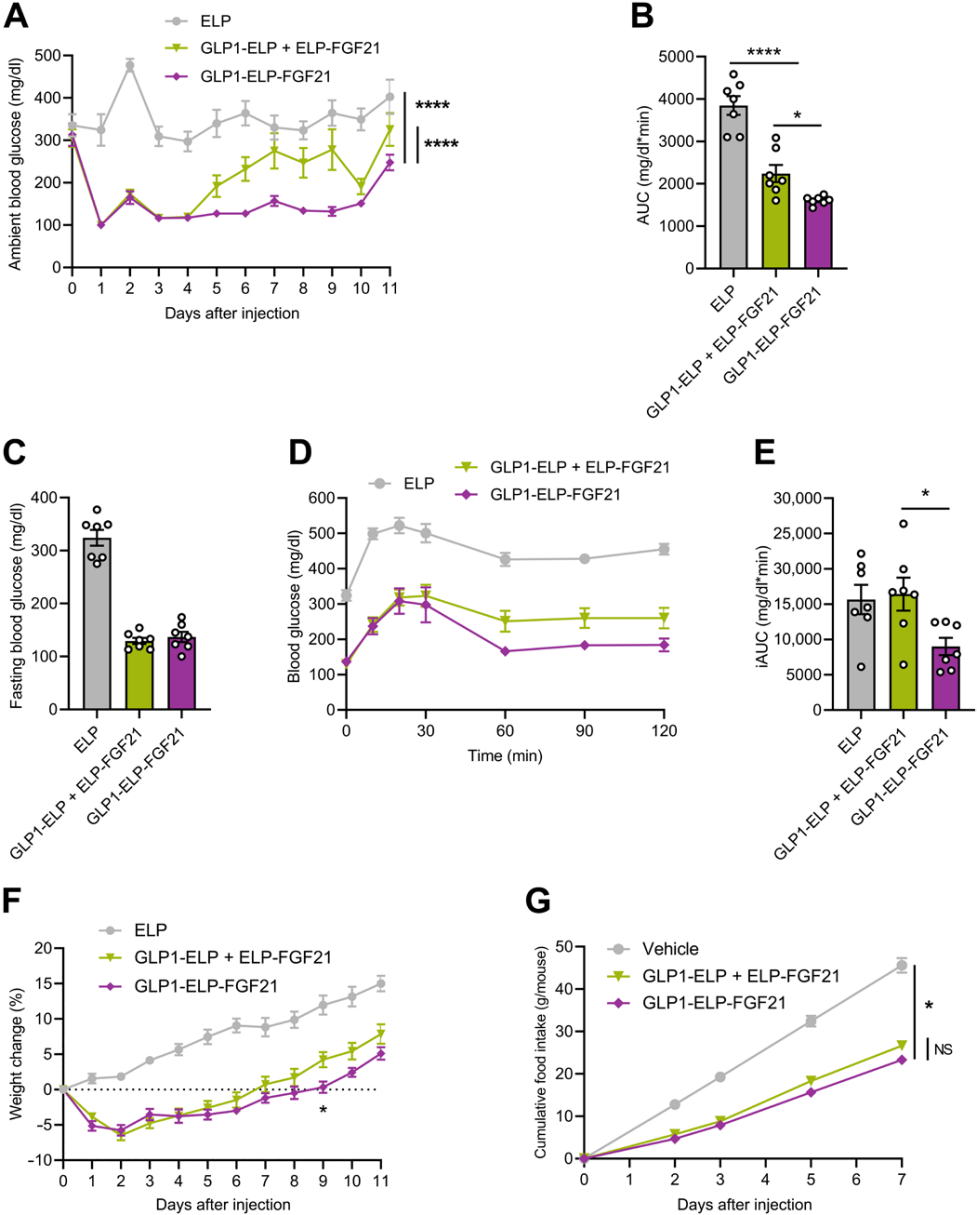
A GLP1-ELP-FGF21 dual-agonist format is therapeutically preferable to a GLP-1/FGF21 single-agonist mixture. (**A** to **F**) Six-week-old *db/db* mice (*n* = 7) received a single subcutaneous injection of GLP1-ELP-FGF21 (1000 nmol/kg), a 1:1 equimolar mixture of GLP1-ELP and ELP-FGF21, or an ELP-only control. Ambient blood glucose levels were measured every 24 hours until animals returned to baseline levels and are reported as raw values (A) or blood glucose versus time AUC (B). (C to E) Glucose challenge. Six days after treatment, mice were fasted 5 hours, baseline blood glucose levels were measured (C), and mice were injected intraperitoneally with glucose (0.75 g/kg). Blood glucose levels were measured at indicated time points (D), and blood glucose versus time incremental AUC (iAUC) values were calculated (E). Body weights were recorded daily and are reported as a percentage change from preinjection weight over time (F). (**G**) Six-week-old *db/db* mice (*n* = 6 to 7) received a single subcutaneous injection of GLP1-ELP-FGF21 (1000 nmol/kg), a 1:1 equimolar mixture of GLP1-ELP and ELP-FGF21, or vehicle control. Food consumption was measured every 1 to 2 days and is reported as cumulative food intake per mouse. Data are presented as means ± SEM and were analyzed by one-way ANOVA or two-way repeated-measures ANOVA, followed by Dunnett’s tests. *P < 0.05 and ****P < 0.0001.

Ambient blood glucose levels following GLP1-ELP-FGF21 treatment were reduced from >300 mg/dl and sustained at <150 mg/dl for ~10 days (Fig. 5A). These data confirm previously observed effects at the same dose (Fig. 3B) and are consistent with therapeutic drug levels maintained in the plasma out to ~10 days, as estimated from the dual-agonist pharmacokinetic profile (Fig. 3E). In contrast, the 1:1 mixture cohort maintained blood glucose at <150 mg/dl for only 4 days, after which the levels started returning to baseline (Fig. 5A). These results are consistent with the 1:1 mixture pharmacokinetic data, which show the plasma concentration of ELP-FGF21 dropping below the estimated minimal therapeutic level of 100 nm after day 4 (Fig. 4A), suggesting a crucial role for FGF21 in the combination treatment’s ability to maintain maximal glycemic control. AUC analysis of the ambient blood glucose versus time data confirmed that the GLP1-ELP-FGF21 treatment afforded significantly superior glucose control compared to the 1:1 mixture (Fig. 5B).

Fasting blood glucose levels measured before the GTT on day 6 were potently reduced upon GLP-1/FGF21 combination treatment and to an equivalent degree regardless of drug format (136.9 ± 9.6 mg/dl for GLP1-ELP-FGF21 and 129.3 ± 5.8 mg/dl for the GLP1-ELP + ELP-FGF21 mixture) (Fig. 5C). However, when compared to the day-6 ambient blood glucose levels (127.1 ± 4.7 mg/dl for GLP1-ELP-FGF21 and 232.4 ± 27.9 mg/dl for the GLP1-ELP + ELP-FGF21 mixture) (Fig. 5A), it is clear that glycemic control has started to wear off in the mixture-treated group at this time, whereas the dual-agonist cohort remains in the normoglycemic range regardless of fed versus fasted state. The ELP control displayed ambient and fasting glucose levels of 364.1 ± 29.0 and 324.1 ± 15.1 mg/dl, respectively, on day 6 (Fig. 5, A and C), demonstrating that the ELP alone, as expected, had no therapeutic activity.

While both the dual-agonist and the single-agonist mixture treatment cohorts displayed improved glucose tolerance (Fig. 5D), an incremental AUC (iAUC) analysis, which sets the baseline for each animal to its *t* = 0 blood glucose, revealed a significant reduction in the dual-agonist group only (Fig. 5E). Mice treated with GLP1-ELP-FGF21 recovered to near-baseline levels within 60 min (Fig. 5D), whereas the animal cohorts treated with the 1:1 mixture or the ELP did not return to their respective baselines following a glucose excursion. These results clearly demonstrate the robust degree of glucose tolerance that is exclusive to the dual-agonist formulation.

Both combination therapy formats induced weight loss—reducing body weights by 5.8 ± 0.8 and 6.5 ± 0.7% for the dual agonist and 1:1 mixture cohorts, respectively (Fig. 5F). GLP1-ELP-FGF21 inhibited weight gain to a greater degree, maintaining a net weight loss effect through day 8, while treatment with the 1:1 mixture maintained a net weight loss effect through only day 6 (Fig. 5F). These results further support the delivery of GLP-1 and FGF21 as a single molecule, as data point to a more consistent modulation of drug release into the systemic circulation. When food intake was tracked over 7 days following treatment, the dual-agonist and 1:1 mixture cohorts consumed chow at equivalent rates (Fig. 5G), indicating that differences in body weight gain were dependent on energy utilization and not to differential effects on satiety.

In summary, these findings clearly show that GLP-1 and FGF21 act synergistically in diabetic mice. Furthermore, fusion of GLP-1 and FGF21 to an ELP to create a depot-forming dual agonist results in a superior therapeutic outcome compared to a mixture of the two respective drugs, validating a multiagonist approach for the GLP-1/FGF21 combination therapy.

### A GLP1-ELP-FGF21 dual agonist does not increase risk of hypoglycemia

Administering GLP-1 and FGF21 in combination was not expected to pose a risk of hypoglycemia, as each respective agonist is believed to contain built-in safety mechanisms preventing dangerous dips in blood glucose. FGF21 lowers glucose levels primarily by increasing insulin sensitivity, while GLP-1 stimulates insulin secretion only in the presence of elevated glucose (*7*, *32*). The dual agonist was nevertheless tested for increased risk of hypoglycemia during prolonged fasting (nocturnal hypoglycemia) and following recovery from a prandial glucose spike (reactive hypoglycemia) (*33*, *34*). *Db/db* mice were treated with GLP1-ELP-FGF21 (1000 nmol/kg) or vehicle, and ad libitum fed blood glucose levels measured 48 hours after treatment showed significant reductions in the dual-agonist cohort, as anticipated (fig. S2C). Mice were then subjected to an overnight 16-hour fast, after which both cohorts maintained blood glucose levels above the conventionally defined threshold of hypoglycemia, 50 to 55 mg/dl (fig. S2C) (*33*), indicating that dual agonist–treated mice tolerated the extended fast. Furthermore, during the recovery phase from *t* = 60 to *t* = 120 min following a glucose challenge, blood glucose levels in the GLP1-ELP-FGF21 cohorts returned to near preinjection baselines, ~150 mg/dl, without evidence of hypoglycemia (Figs. 4C and 5D). Thus, dual-agonist treatment did not appear to increase susceptibility to reactive hypoglycemia due to excessive insulin secretion.

## DISCUSSION

The GLP-1 system has been tractable as a therapeutic target, and peptide agonists of the GLP-1R are now well established in clinical practice. However, the enormous promise suggested by early studies (*35*, *36*) has not been realized, as GLP-1RAs have potencies only slightly greater than conventional antidiabetes agents. We are now seeing a shift toward GLP-1RA–based combination therapies designed to simultaneously target (i) glucose metabolism, (ii) food intake, and (iii) energy expenditure for effective treatment of metabolic diseases (*37*). Recent insights into structure-activity relationships have led to the development of GLP-1–based chimeras that incorporate two or more receptor agonist properties into a hybrid peptide sequence. These coagonist molecules appear to amplify the effects of GLP-1 while broadening its therapeutic window (*38*) and are now moving into clinical trials (*37*, *39*). However, by necessity, coagonist drugs incorporate only the action of peptides with sequence and receptor similarities and are therefore limited to the glucagon superfamily, which have overlap in downstream signaling (*37*).

We describe the fusion of GLP-1, an incretin peptide that binds to and activates its cognate receptor on pancreatic β cells to stimulate insulin release, with FGF21, a 20-kDa protein that activates the receptor tyrosine kinase receptor class and is structurally quite dissimilar from the glucagon peptide superfamily (*40*). The fusion of GLP-1 with FGF21 was motivated by their complementary pharmacology, thereby addressing the three criteria for an ideal combination therapy to treat metabolic disease: improved glucose metabolism, decreased food intake, and enhanced energy expenditure. This pairing was further supported by evidence that FGF21 mediates the therapeutic responses to both GLP-1 (*41*) and to the recently used GLP-1 coagonist, glucagon (*42*).

As a hybrid peptide design was not feasible here, a recombinant fusion strategy was used to incorporate GLP-1 and FGF21 into a single biomolecule. A heterodimeric Fc fusion is one approach that has been previously used for the tandem display of two distinct peptides (*43*) and offers the advantage of recombinant synthesis. An Fc fusion–based dual-agonist architecture, however, stipulates that each protein be fused at the same terminus. This technology therefore cannot be applied to protein drug pairs that require opposite exposed termini for proper function—such as GLP-1 and FGF21—which require exposed N and C termini, respectively, for biological activity. Thus we combined GLP-1 and FGF21 as a head-to-tail fusion and selected an ELP as the linker between them.

The choice of a genetically encoded ELP, rather than a synthetic polymer linker such as polyethylene glycol (*44*), was driven by four considerations. First, we hypothesized that a long and unstructured linker would provide the length and flexibility necessary to prevent steric hindrance between GLP-1 and FGF21, thereby allowing the two drugs to bind their respective targets on the same or adjacent cells. ELPs are intrinsically disordered and, hence, unstructured and flexible biomolecules that satisfy the structural requirements for the linker. Second, the genetically encoded GLP1-ELP-FGF21 dual-agonist design allows recombinant expression of the resulting polypeptide fusion. Third, the fusion retains the reversible soluble-insoluble LCST phase transition behavior of the ELP and is thus easily purified by a nonchromatographic method—ITC, which will greatly simplify the manufacturing of this drug at the scale necessary for clinical translation. Fourth, considering that most peptide-based drugs—including GLP-1 and FGF21—are pharmacologically limited by their short plasma half-lives, the ELP was designed to phase transition at body temperature such that the fusion formed a depot under the skin upon subcutaneous injection. Because of the inverse dependence of *T*_t_ as a function of concentration, the ELP depot dissolves upon injection from the margins to the core, releasing soluble GLP1-ELP-FGF21 molecules into systemic circulation and thereby providing an injectable platform for sustained delivery of the dual-agonist drug. The consequence of this design feature is a long-circulating biologic for the treatment of T2D that exerts its pharmacological activity for up to 10 days following a single subcutaneous injection.

This report describing the incorporation of GLP-1 and FGF21 into a dual-agonist drug is, to our knowledge, the first publication revealing the impressively potent effects of this complementary combination. The GLP1-ELP-FGF21 treatment facilitates greater weight reduction and enhanced glycemic control compared to GLP-1RA therapy in a mouse model plagued by profound defects in energy and glucose regulation. We hypothesize that the improved glucose tolerance and augmented weight reduction observed in the dual-agonist cohort are due to increased insulin sensitivity and thermogenic action mediated specifically by the FGF21 component. Furthermore, when tested alongside a 1:1 mixture of long-acting GLP-1 and FGF21 single agonists, our dual-agonist drug showed consistently superior metabolic effects. We attribute GLP1-ELP-FGF21’s enhanced efficacy to the finding that therapeutic levels of each drug component are maintained for a larger portion of the dosing cycle when GLP-1 and FGF21 are delivered as a single molecule compared to a drug mixture.

Our dual-agonist design for protein-based therapeutics also provides the advantage of easier administration. Clinical applications of injectables are constrained by volume, viscosity, and patient pain tolerance. While smaller needle diameters are correlated with ease of injection and increased patient comfort, the viscosity of the injected solution is limited by the maximum injection force that the subcutaneous tissue can tolerate without damage or inflammation (*45*, *46*). Furthermore, high concentrations can promote aggregation of protein-based drugs during long-term storage (*46*). Solution viscosity can be reduced through dilution; however, subcutaneous injection volumes exceeding 1.5 to 2.5 ml are once again associated with discomfort and injection site tissue damage (*46*). Delivering two agonists on one ELP—or any polymer-based delivery scaffold—reduces the polymer stoichiometry by 50% when compared to delivering each respective agonist on its own scaffold. Thus, a unimolecular dual-agonist approach to drug design reduces required injection concentration and viscosity, resulting in a more readily injectable therapeutic with superior stability.

In conclusion, the findings presented here reveal the potent antiobesity and antidiabetic effects that result when GLP-1 is delivered in combination with FGF21 as a long-circulating and long-acting fusion, suggesting that a sustained release formulation of a GLP-1/FGF21 dual agonist may have great potential for the treatment of overweight patients with T2D. We hope that these studies inspire further investigation into the mechanisms underlying these profound effects. Considering that the biology of FGF21is distinct in humans from both rodents and primates (*47*), human clinical testing of this combination will be particularly germane. In addition to designing a drug with dual GLP-1/FGF21 receptor agonism, our results also lay the groundwork for engineering an innovative class of multiagonist drugs that incorporate sustained release modules alongside activators for distinct receptor signaling networks, allowing for a broader range of metabolic applications.

## MATERIALS AND METHODS

### Experimental design

#### Research objective

The purpose of this set of studies was to design, produce, and characterize a unimolecular drug with dual agonism for the GLP-1 and FGF21 receptors and an ELP linker to serve as a delivery module. We hypothesized that, should the fusion successfully express, we could harness the ELP properties for chromatography-free purification as well as a depot-based delivery platform. On the basis of previous work with single-agonist ELP fusions, we hypothesized that coexpression of GLP-1 and FGF21 on an ELP would retain biological activity in each respective agonist. Furthermore, we predicted that GLP-1 and FGF21 would act cooperatively for superior efficacy in a rodent model of T2D and obesity.

#### Sample size

Sample sizes were chosen on the basis of the scatter in data previously collected by our research group in the same mouse model (*db/db*) treated with ELP depots (*17*, *18*). An *N* = 6 was deemed sufficient for detecting significance (power = 0.8, α = 0.05, two-tailed) between treatment and control groups, and thus, an *N* = 6 to 7 was chosen for efficacy studies. An *N* = 4 was deemed sufficient for pharmacokinetic and in vivo dose-response studies, as these studies were intended to identify trends. Note that one animal in the dose-response cohort (250 mg/kg) was removed from the study due to injuries sustained from fighting. In addition, note that food intake data used an *N* = 2, as food consumption was monitored for each cage of three to four mice, with two cages per treatment. A higher *N* could have been achieved by single housing the animals; however, our institutional animal care committee discourages long-term single housing of mice.

#### Rules for stopping data collection

GLP-1 analogs administered at high doses have been known to cause nausea and gastrointestinal discomfort in humans. Thus, any mice showing signs of distress (hunching, ruffled fur, loss of fur, and slow movement), losing more than 15% body weight, or exhibiting blood glucose levels in the hypoglycemic range (<50 mg/dl) were to be removed from the study. Note that the antidiabetic drugs used in these studies were not expected to pose a risk for hypoglycemia.

#### Exclusion criteria

Ambient blood glucose levels were measured in mice three consecutive days before study initiation. Any untreated mouse that exhibited a blood glucose reading above the range of the glucometer (>600 mg/dl) was prospectively excluded from the study.

#### Selection of endpoints

The dose-response study was carried out until all ambient blood glucose levels started returning to baselines. All pharmacokinetic data plots were analyzed out to day 16, as this is the time point at which the single-agonist control reached the lower limit of detection on the gamma counter. The long-term in vivo efficacy study was carried out for 4 weeks, as this duration is sufficient for observable changes in %HbA1c levels.

#### Replicates

Turbidity scan and in vitro dose-response experiments were each carried out two to three times with consistent results. In vivo efficacy of the dual-agonist drug was confirmed in three independent studies.

#### Randomization

Before in vivo study initiation, ambient blood glucose levels were measured in mice on days −3, −2, and − 1, and body weights were measured on day −1. Mice were then randomized into control and treatment groups using random.org while stratifying to maintain consistent prestudy mean baseline glucose levels and body weights in each cohort. All studies aside from the long-term efficacy study used a random grouping of treatment and control mice in cages. To assess food intake, the long-term efficacy study required that treatment cohorts be housed together. For all in vivo studies, cages and mice were identified by numbers and/or letters and not by treatment to maintain a level of blinding between technician and experimental design.

### Expression vector synthesis

The nucleotide sequence encoding the 182–amino acid murine wild-type FGF21 protein, minus the signal peptide, was codon optimized for *E. coli* expression and ligated into a pET-24a+ vector modified for seamless fusion of genes (*48*). Point mutations for amino acid substitutions L99R, P172G, and L173S were introduced to enhance protein stability, as previously described (*18*), and the mutated *Fgf21* gene was fused at the 5′ end to a gene encoding an ELP, following a previously reported seamless cloning strategy (*48*). The ELP consisted of 120 repeats of a (Val-Pro-Gly-X_aa_-Gly) pentapeptide, where X_aa_ signifies a 4:1 ratio of Val:Ala, and the final vector encoded the polypeptide fusion “ELP-FGF21.”

The rationale for the design of the GLP-1 analog sequence has been previously described (*24*). The analog consists of GLP-1 (7-37) with A8G, G22E, and R36A amino acid substitutions, as well as an AA leader at the N terminus to enable activation through cleavage by DPP4. Following a similar process as for *Fgf21*, the nucleotide sequence encoding the resulting 32–amino acid GLP-1 peptide was codon-optimized for *E. coli* expression, ligated into the modified pET-24a+ vector, and fused at the 3′ end to the gene encoding the ELP described above. The final vector encoded the polypeptide fusion “GLP1-ELP.” For synthesis of the vector encoding the GLP-1/FGF21 dual-agonist drug, the gene encoding GLP1-ELP was fused at the 3′ end to the mutated *Fgf21* gene following a seamless cloning strategy (*48*), and the final vector encoded the polypeptide fusion referred to as GLP1-ELP-FGF21.

### Protein expression and purification

For the production of GLP1-ELP, the GLP1-ELP–encoding expression vector was transformed into Ultra BL21(DE3) cells (Edge BioSystems, Gaithersburg, MD), and protein expression and purification from the soluble fraction of cell lysate by ITC were carried out as described previously (*17*). ELP-FGF21– and GLP1-ELP-FGF21–encoding expression vectors were transformed into SHuffle cells (New England Biolabs), and modified methods for expression and ITC were used as follows.

A starter culture containing 50 ml of terrific broth (TB; 55 g/liter) and 250 μM kanamycin was inoculated and grown overnight at 37°C with orbital shaking at 250 rpm. The starter culture was centrifuged, resuspended in TB, and used to inoculate three 1-liter volumes of TB and kanamycin in 6-liter Erlenmeyer flasks. The flasks were cultured at 30°C with orbital shaking at 200 rpm until they reached an OD_600_ of 2.0. Protein expression was then induced by addition of 250 μM isopropyl-β-d-thiogalactopyranoside. The culturing temperature was reduced to 16°C, and growth was allowed to proceed for an additional 18 hours.

Bacterial cultures were centrifuged at 4°C for 10 min at 3365 “x g” (units of g force) and resuspended in cold phosphate-buffered saline (PBS). Cell membranes were disrupted via sonication (Q500 sonicator, QSonica, Newtown, CT) and pulsed at 10-s on and 40-s off for a total sonication time of 90 s. DNA was precipitated by addition of 10% polyethyleneimine, and cell lysates were separated into soluble and insoluble fractions by centrifugation at 4°C for 10 min at 23,645 “x g” (units of g force). The soluble fraction was brought to room temperature, and the ELP fusion protein was purified from solution by ITC. In this process, the phase transition of the ELP fusion protein was triggered by addition of 0.2 M (NH_4_)_2_SO_4_, producing a turbid suspension due to coacervation of the ELP fusion. The suspension was centrifuged at 25°C for 15 min at 23,426 “x g” (units of g force); this step is referred to as a “hot spin.” The supernatant was discarded, and the pellet was resolubilized in PBS at 4°C with 25 rpm gentle rotation (R4045 RotoBot Programmable Rotator, Benchmark Scientific, Sayreville, NJ). The resulting solution was centrifuged at 4°C for 5 min at 18,407 “x g” (units of g force) to pellet insoluble contaminants, and the supernatant was reserved; this step is referred to as a “cold spin.”

The ITC process was repeated by warming the solution to room temperature, adding (NH_4_)_2_SO_4_ to trigger the phase transition, centrifuging at 25°C for 8 min at 18,407 “x g” (units of g force) to pellet the ELP fusion protein, resolubilizing the pellet in PBS at 4°C with gentle rotation, and centrifuging at 4°C for 5 min at 18,407 “x g” (units of g force). Three total rounds of ITC were necessary to isolate the fusion from contaminants, and the final products were visualized on a Coomassie- or CuCl_2_-stained SDS-PAGE gel.

### Endotoxin purification and testing

All proteins were endotoxin-purified using Acrodisc units (Pall Corporation, Port Washington, NY), and resulting endotoxin levels were tested using the Endosafe nexgen-PTS spectrophotometer (Charles River Laboratories, Wilmington, MA).

### Extracellular signal–regulated kinase phosphorylation assay

For a quantitative evaluation of FGF21 in vitro activity, a human embryonic kidney (HEK) 293 cell line was previously generated that stably expresses murine β-klotho and FGF receptor 1 (FGFR1) (*18*) and thereby enables FGF21-mediated extracellular signal–regulated kinase 1/2 (ERK1/2) phosphorylation. Cells were seeded at 5 × 10^4^ cells/cm^2^ and adhered overnight. After serum starvation for 6 hours, cells were treated with serial dilutions of FGF21-containing fusion proteins or native mouse FGF21 (ProSpec-Tany, East Brunswick, NJ) for 5 min. Cells were lysed and assessed for phospho-ERK1/2 and total ERK1/2 content using the AlphaLISA SureFire Ultra Assay Kits (PerkinElmer) and the EnSpire Alpha Plate Reader (PerkinElmer). Phospho-ERK1/2 was normalized to total ERK1/2 and fit to a three-parameter dose-response curve to determine EC_50_ values using GraphPad Prism 8 software (La Jolla, CA).

### Adenosine 3′,5′-monophosphate production assay

GLP-1 in vitro activity was quantified by a cell-based assay that uses a HEK293 cell line that stably expresses the GLP-1R and an adenosine 3′,5′-monophosphate (cAMP)–inducible luciferase reporter (*49*). Cells were seeded at 1 × 10^5^ cells/cm^2^ into 96-well plates and adhered overnight. Concurrently, GLP-1–containing fusions were incubated overnight at 4°C with DPP4 (ProSpec-Tany) at a 1:500 DPP4:GLP-1 molar ratio to cleave the AA leader and expose the active N terminus of GLP-1. In the morning, cell medium was replaced with induction buffer (129 mM NaCl, 4.8 mM KCl, 1.2 mM MgSO_4_, 1.2 mM KH_2_PO_4_, 2.5 mM CaCl_2_, 5 mM NaHCO_3_, 10 mM Hepes, 0.5% bovine serum albumin, and 50 μM 3-isobutyl-1-methylxanthine). Cells were treated with serial dilutions of GLP-1–containing fusion proteins or native human GLP-1 (7-37) (ProSpec-Tany) in induction buffer for 5 hours, at which point supernatants were removed and replaced with Bright-Glo (Promega, Madison, WI). Luminescence of the supernatant samples was measured on a Victor X3 plate reader (PerkinElmer) and normalized to zero drug treatment control wells. Data were fit to a three-parameter dose-response curve to determine EC_50_ values using the GraphPad Prism 8 software.

### Phase behavior characterization

The LCST phase transition behavior of ELP fusion proteins was evaluated by monitoring the OD_350_ of solutions in PBS as a function of temperature on a Cary 300 ultraviolet-visible spectrophotometer equipped with a multicell thermoelectric temperature controller (Agilent Technologies, Santa Clara, CA). Heating and cooling were set to a rate of 1°C/min. The *T*_t_ was defined as the temperature at which the OD reached 50% of its maximal value (*21*).

### Animals

In vivo studies were conducted in accordance with the AAALAC International–accredited Duke Institutional Animal Care and Use Committee. Five-week-old B6.BKS(D)-Lepr^db^/J (“db/db”) male mice were purchased from the Jackson laboratory (Bar Harbor, ME) and maintained on a 12-hour/12-hour light/dark cycle with ad libitum access to food (LabDiet 5053) and water. Animals were group-housed and allowed a 1-week acclimation before study initiation.

### ELP fusion treatments

ELP fusion proteins were administered at 150 to 200 μM concentration via injection into the subcutaneous space on the hind flank. Animals received either a single subcutaneous injection for short-term studies or weekly subcutaneous injections for chronic studies. The “1:1 mixture” treatment group received a single injection containing an equimolar mixture of GLP1-ELP and ELP-FGF21, with the indicated dose referring to the dose of each respective fusion in the mixture. Vehicle control refers to PBS.

### Ambient blood glucose measurements

Blood glucose levels were measured from a tail vein puncture with the AlphaTRAK 2 Blood Glucose Meter (Zoetis, Parsippany-Troy Hills, NJ) every 24 hours, 1 to 2 hours following onset of the light cycle. Data are presented either as raw values or as a magnitude change from a mean baseline established from three independent measurements collected before treatment. AUC was calculated with the GraphPad Prism 8 software using the trapezoidal rule and setting a *Y* = 0 baseline. Treatment group AUCs were normalized to the vehicle-treated group where indicated.

### Glucose tolerance test

Animals were fasted at the onset of the light cycle for 5 hours with ad libitum access to water and then intraperitoneally injected with d-glucose (0.75 g/kg). Blood glucose levels were measured from a tail snip with the Contour Blood Glucose Meter (Bayer, Leverkusen, Germany) at *t* = 0 (before glucose injection), 10, 20, 30, 60, 90, and 120 min. AUCs were calculated as described for ambient blood glucose measurements, with iAUC using a baseline equal to each respective individual’s *t* = 0 measurement.

### Extended fast blood glucose measurements

Animals were fasted for 16 hours starting at 4:00 p.m. with ad libitum access to water, after which blood glucose levels were measured from the tail vein as described for ambient blood glucose measurements.

### Blood parameters

Five microliters of blood samples were collected from the tail veins, and %HbA1c was measured from whole blood using a DCA Vantage analyzer (Siemens).

### Food intake

For food intake studies, animals were group-housed four per cage, and food pellets were weighed every 1 to 2 days. Total cage food intake was calculated for each time increment, averaged per animal, and summed over the course of the study to yield a cumulative food intake per mouse. *N* = 8 treatment groups were divided into two cages, allowing for an *N* = 2 food intake SE calculation.

### Pharmacokinetic studies

Tyrosine residues on GLP1-ELP, ELP-FGF21, or GLP1-ELP-FGF21 fusion proteins were reacted with Na^125^I radionuclide (PerkinElmer) using Pierce Pre-Coated Iodination Tubes (Thermo Fisher Scientific) and the indirect method for iodination. Radiolabeled protein was purified from unreacted radionuclide with Zeba Spin Desalting Columns (Thermo Fisher Scientific). Activities of radiolabeled constructs were measured with the Atomlab 400 Dose Calibrator (Biodex, Shirley, NY) and correlated to protein concentration. Mice received a single subcutaneous injection of radiolabeled fusion, and 10 μl of blood samples were collected at frequent time points from the tail vein and stored at room temperature until radioactivity quantification. Sample counts were measured at the end of the study on a Wallac Wizard 1480 automatic gamma counter (PerkinElmer). An activity-versus-count standard curve was used to convert sample counts to activities and subsequently to moles of drug.

### Pharmacokinetic analysis

The *C*_max_ was recorded as observed, as well as time to reach *C*_max_ (*t*_max_). AUC was estimated using a serum concentration of 0 nM at time zero and extrapolated to 16 days after administration based on a linear regression curve fit to the terminal portion of the log serum concentration versus time curve. Absorption half-life (*t*_1/2,abs_) was estimated from the slope of the linear regression curve. When a drug administered at an extravascular site yields a terminal half-life greater than that resulting from an intravenous bolus, the terminal half-life reflects the absorption half-life (*29*).

### Statistical analysis

Data are presented as means ± SEM. Normally distributed data were analyzed by ordinary one-way analysis of variance (ANOVA), followed by Dunnett’s multiple comparisons tests, or by two-way repeated-measures ANOVA, followed by Dunnett’s, Tukey’s, or Sidak’s tests. In vitro EC_50_ values were compared by one-way ANOVA followed by Tukey’s tests.

## Supplementary Material

aaz9890_SM.pdf

## SUPPLEMENTARY MATERIALS

Supplementary material for this article is available at http://advances.sciencemag.org/cgi/content/full/6/35/eaaz9890/DC1

## Appendix

View/request a protocol for this paper from *Bio-protocol*.

## References

[1] Centers for Disease Control and Prevention, “National diabetes statistics report, 2020,” (Atlanta, 2020).

[2] U.K. Prospective Diabetes Study Group, U.K. prospective diabetes study 16: Overview of 6 years’ therapy of type II diabetes: A progressive disease. Diabetes 44, 1249–1258 (1995). 7589820

[3] Machado-Alba J. E., Machado-Duque M. E., Moreno-Gutierrez P. A., Time to and factors associated with insulin initiation in patients with type 2 diabetes mellitus. Diabetes Res. Clin. Pract. 107, 332–337 (2015). 25648389 10.1016/j.diabres.2015.01.018

[4] Ringborg A., Lindgren P., Yin D. D., Martinell M., Stålhammar J., Time to insulin treatment and factors associated with insulin prescription in Swedish patients with type 2 diabetes. Diabetes Metab. 36, 198–203 (2010). 20347376 10.1016/j.diabet.2009.11.006

[5] American Diabetes Association, Standards of medical care in diabetes—2020. Diabetes Care 43, S1–S212 (2020).31862741

[6] Baggio L. L., Drucker D. J., Biology of incretins: GLP-1 and GIP. Gastroenterology 132, 2131–2157 (2007). 17498508 10.1053/j.gastro.2007.03.054

[7] Meier J. J., GLP-1 receptor agonists for individualized treatment of type 2 diabetes mellitus. Nat. Rev. Endocrinol. 8, 728–742 (2012). 22945360 10.1038/nrendo.2012.140

[8] Davies M. J., D’Alessio D. A., Fradkin J., Kernan W. N., Mathieu C., Mingrone G., Rossing P., Tsapas A., Wexler D. J., Buse J. B., Management of hyperglycemia in type 2 diabetes, 2018. A consensus report by the American Diabetes Association (ADA) and the European Association for the Study of Diabetes (EASD). Diabetes Care 41, 2669–2701 (2018). 30291106 10.2337/dci18-0033 PMC6245208

[9] Brandt S. J., Müller T. D., DiMarchi R. D., Tschöp M. H., Stemmer K., Peptide-based multi-agonists: A new paradigm in metabolic pharmacology. J. Intern. Med. 284, 581–602 (2018). 30230640 10.1111/joim.12837

[10] Finan B., Yang B., Ottaway N., Smiley D. L., Ma T., Clemmensen C., Chabenne J., Zhang L., Habegger K. M., Fischer K., Campbell J. E., Sandoval D., Seeley R. J., Bleicher K., Uhles S., Riboulet W., Funk J., Hertel C., Belli S., Sebokova E., Conde-Knape K., Konkar A., Drucker D. J., Gelfanov V., Pfluger P. T., Müller T. D., Perez-Tilve D., DiMarchi R. D., Tschöp M. H., A rationally designed monomeric peptide triagonist corrects obesity and diabetes in rodents. Nat. Med. 21, 27–36 (2014). 25485909 10.1038/nm.3761

[11] Sandoval D. A., D'Alessio D. A., Physiology of proglucagon peptides: Role of glucagon and GLP-1 in health and disease. Physiol. Rev. 95, 513–548 (2015). 25834231 10.1152/physrev.00013.2014

[12] Kharitonenkov A., Shiyanova T. L., Koester A., Ford A. M., Micanovic R., Galbreath E. J., Sandusky G. E., Hammond L. J., Moyers J. S., Owens R. A., Gromada J., Brozinick J. T., Hawkins E. D., Wroblewski V. J., Li D.-S., Mehrbod F., Jaskunas S. R., Shanafelt A. B., FGF-21 as a novel metabolic regulator. J. Clin. Invest. 115, 1627–1635 (2005). 15902306 10.1172/JCI23606 PMC1088017

[13] Kliewer S. A., Mangelsdorf D. J., A dozen years of discovery: Insights into the physiology and pharmacology of FGF21. Cell Metab. 29, 246–253 (2019). 30726758 10.1016/j.cmet.2019.01.004 PMC6368396

[14] Gaich G., Chien J. Y., Fu H., Glass L. C., Deeg M. A., Holland W. L., Kharitonenkov A., Bumol T., Schilske H. K., Moller D. E., The effects of LY2405319, an FGF21 analog, in obese human subjects with type 2 diabetes. Cell Metab. 18, 333–340 (2013). 24011069 10.1016/j.cmet.2013.08.005

[15] Talukdar S., Zhou Y., Li D., Rossulek M., Dong J., Somayaji V., Weng Y., Clark R., Lanba A., Owen B. M., Brenner M. B., Trimmer J. K., Gropp K. E., Chabot J. R., Erion D. M., Rolph T. P., Goodwin B., Calle R. A., A long-acting FGF21 molecule, PF-05231023, decreases body weight and improves lipid profile in non-human primates and type 2 diabetic subjects. Cell Metab. 23, 427–440 (2016). 26959184 10.1016/j.cmet.2016.02.001

[16] Sanyal A., Charles E. D., Neuschwander-Tetri B. A., Loomba R., Harrison S. A., Abdelmalek M. F., Lawitz E. J., Halegoua-DeMarzio D., Kundu S., Noviello S., Luo Y., Christian R., Pegbelfermin (BMS-986036), a PEGylated fibroblast growth factor 21 analogue, in patients with non-alcoholic steatohepatitis: A randomised, double-blind, placebo-controlled, phase 2a trial. Lancet 392, 2705–2717 (2018). 30554783 10.1016/S0140-6736(18)31785-9

[17] Luginbuhl K. M., Schaal J. L., Umstead B., Mastria E. M., Li X., Banskota S., Arnold S., Feinglos M., D’Alessio D., Chilkoti A., One-week glucose control via zero-order release kinetics from an injectable depot of glucagon-like peptide-1 fused to a thermosensitive biopolymer. Nat. Biomed. Eng. 1, 0078 (2017). 29062587 10.1038/s41551-017-0078 PMC5650111

[18] Gilroy C. A., Roberts S., Chilkoti A., Fusion of fibroblast growth factor 21 to a thermally responsive biopolymer forms an injectable depot with sustained anti-diabetic action. J. Control. Release 277, 154–164 (2018). 29551712 10.1016/j.jconrel.2018.03.015 PMC5945213

[19] Urry D. W., Free energy transduction in polypeptides and proteins based on inverse temperature transitions. Prog. Biophys. Mol. Biol. 57, 23–57 (1992). 1549698 10.1016/0079-6107(92)90003-o

[20] Chilkoti A., Christensen T., MacKay J. A., Stimulus responsive elastin biopolymers: Applications in medicine and biotechnology. Curr. Opin. Chem. Biol. 10, 652–657 (2006). 17055770 10.1016/j.cbpa.2006.10.010 PMC3732176

[21] Urry D. W., Physical chemistry of biological free energy transduction as demonstrated by elastic protein-based polymers. J. Phys. Chem. B 101, 11007–11028 (1997).

[22] Meyer D. E., Chilkoti A., Purification of recombinant proteins by fusion with thermally-responsive polypeptides. Nat. Biotechnol. 17, 1112–1115 (1999). 10545920 10.1038/15100

[23] Urry D. W., Luan C. H., Parker T. M., Gowda D. C., Prasad K. U., Reid M. C., Safavy A., Temperature of polypeptide inverse temperature transition depends on mean residue hydrophobicity. J. Am. Chem. Soc. 113, 4346–4348 (1991).

[24] Amiram M., Luginbuhl K. M., Li X., Feinglos M. N., Chilkoti A., A depot-forming glucagon-like peptide-1 fusion protein reduces blood glucose for five days with a single injection. J. Control. Release 172, 144–151 (2013). 23928357 10.1016/j.jconrel.2013.07.021 PMC3834218

[25] Micanovic R., Raches D. W., Dunbar J. D., Driver D. A., Bina H. A., Dickinson C. D., Kharitonenkov A., Different roles of N- and C- termini in the functional activity of FGF21. J. Cell. Physiol. 219, 227–234 (2009). 19117008 10.1002/jcp.21675

[26] Hassouneh W., Christensen T., Chilkoti A., Elastin-like polypeptides as a purification tag for recombinant proteins. Curr. Protoc. Protein Sci. 61, 6.11.11–6.11.16 (2010). 10.1002/0471140864.ps0611s61 PMC3076942 20814933

[27] Trammell R. A., Cox L., Toth L. A., Markers for heightened monitoring, imminent death, and euthanasia in aged inbred mice. Comp. Med. 62, 172–178 (2012). 22776049 PMC3364702

[28] Meyer D. E., Kong G. A., Dewhirst M. W., Zalutsky M. R., Chilkoti A., Targeting a genetically engineered elastin-like polypeptide to solid tumors by local hyperthermia. Cancer Res. 61, 1548–1554 (2001). 11245464

[29] S. S. Jambhekar, P. J. Breen, *Basic Pharmacokinetics* (Pharmaceutical Press, ed. 2, 2012).

[30] Koenig R. J., Hemoglobin AIc and diabetes mellitus. Annu. Rev. Med. 31, 29–34 (1980). 6994614 10.1146/annurev.me.31.020180.000333

[31] Van Putten L. M., Croon F., The life span of red cells in the rat and the mouse as determined by labeling with DFP^32^ in vivo. Blood 13, 789–794 (1958). 13560578

[32] Kharitonenkov A., DiMarchi R., Fibroblast growth factor 21 night watch: Advances and uncertainties in the field. J. Intern. Med. 281, 233–246 (2016). 27878865 10.1111/joim.12580

[33] Cryer P. E., Davis S. N., Shamoon H., Hypoglycemia in diabetes. Diabetes Care 26, 1902–1912 (2003). 12766131 10.2337/diacare.26.6.1902

[34] Toft-Nielsen M., Madsbad S., Holst J. J., Exaggerated secretion of glucagon-like peptide-1 (GLP-1) could cause reactive hypoglycaemia. Diabetologia 41, 1180–1186 (1998). 9794105 10.1007/s001250051049

[35] Nauck M. A., Kleine N., Ørskov C., Holst J. J., Willms B., Creutzfeldt W., Normalization of fasting hyperglycaemia by exogenous glucagon-like peptide 1 (7-36 amide) in type 2 (non-insulin-dependent) diabetic patients. Diabetologia 36, 741–744 (1993). 8405741 10.1007/BF00401145

[36] Rachman J., Barrow B. A., Levy J. C., Turner R. C., Near-normalisation of diurnal glucose concentrations by continuous administration of glucagon-like peptide-1 (GLP-1) in subjects with NIDDM. Diabetologia 40, 205–211 (1997). 9049482 10.1007/s001250050664

[37] Clemmensen C., Finan B., Müller T. D., DiMarchi R. D., Tschöp M. H., Hofmann S. M., Emerging hormonal-based combination pharmacotherapies for the treatment of metabolic diseases. Nat. Rev. Endocrinol. 15, 90–104 (2019). 30446744 10.1038/s41574-018-0118-x

[38] Capozzi M. E., DiMarchi R. D., Tschöp M. H., Finan B., Campbell J. E., Targeting the incretin/glucagon system with triagonists to treat diabetes. Endocr. Rev. 39, 719–738 (2018). 29905825 10.1210/er.2018-00117 PMC7263842

[39] Frias J. P., Nauck M. A., Van J., Kutner M. E., Cui X., Benson C., Urva S., Gimeno R. E., Milicevic Z., Robins D., Haupt A., Efficacy and safety of LY3298176, a novel dual GIP and GLP-1 receptor agonist, in patients with type 2 diabetes: A randomised, placebo-controlled and active comparator-controlled phase 2 trial. Lancet 392, 2180–2193 (2018). 30293770 10.1016/S0140-6736(18)32260-8

[40] Beenken A., Mohammadi M., The FGF family: Biology, pathophysiology and therapy. Nat. Rev. Drug Discov. 8, 235–253 (2009). 19247306 10.1038/nrd2792 PMC3684054

[41] Lynch L., Hogan A. E., Duquette D., Lester C., Banks A., Clair K. L., Cohen D. E., Ghosh A., Lu B., Corrigan M., Stevanovic D., Maratos-Flier E., Drucker D. J., O’Shea D., Brenner M., iNKT cells induce FGF21 for thermogenesis and are required for maximal weight Loss in GLP1 therapy. Cell Metab. 24, 510–519 (2016). 27593966 10.1016/j.cmet.2016.08.003 PMC5061124

[42] Habegger K. M., Stemmer K., Cheng C., Müller T. D., Heppner K. M., Ottaway N., Holland J., Hembree J. L., Smiley D., Gelfanov V., Krishna R., Arafat A. M., Konkar A., Belli S., Kapps M., Woods S. C., Hofmann S. M., D’Alessio D., Pfluger P. T., Perez-Tilve D., Seeley R. J., Konishi M., Itoh N., Kharitonenkov A., Spranger J., DiMarchi R. D., Tschöp M. H., Fibroblast growth factor 21 mediates specific glucagon actions. Diabetes 62, 1453–1463 (2013). 23305646 10.2337/db12-1116 PMC3636653

[43] Ha J.-H., Kim J.-E., Kim Y.-S., Immunoglobulin Fc heterodimer platform technology: From design to applications in therapeutic antibodies and proteins. Front. Immunol. 7, 394 (2016). 27766096 10.3389/fimmu.2016.00394 PMC5052280

[44] Caliceti P., Veronese F. M., Pharmacokinetic and biodistribution properties of poly(ethylene glycol)–protein conjugates. Adv. Drug Deliv. Rev. 55, 1261–1277 (2003). 14499706 10.1016/s0169-409x(03)00108-x

[45] Mathaes R., Koulov A., Joerg S., Mahler H.-C., Subcutaneous injection volume of biopharmaceuticals—Pushing the boundaries. J. Pharm. Sci. 105, 2255–2259 (2016). 27378678 10.1016/j.xphs.2016.05.029

[46] D. M. Piedmonte, J. H. Gu, S. R. Brych, M. M. Goss, Practical considerations for high concentration protein formulations, in *Challenges in Protein Product Development*, N. W. Warne, H.-C. Mahler, Eds. (Springer International Publishing, 2018), pp. 163–187.

[47] Kharitonenkov A., DiMarchi R., FGF21 revolutions: Recent advances illuminating FGF21 biology and medicinal properties. Trends Endocrinol. Metab. 26, 608–617 (2015). 26490383 10.1016/j.tem.2015.09.007

[48] McDaniel J. R., MacKay J. A., Quiroz F. G., Chilkoti A., Recursive directional ligation by plasmid reconstruction allows rapid and seamless cloning of oligomeric genes. Biomacromolecules 11, 944–952 (2010). 20184309 10.1021/bm901387t PMC2862688

[49] Riedel M. J., Lee C. W. K., Kieffer T. J., Engineered glucagon-like peptide-1-producing hepatocytes lower plasma glucose levels in mice. Am. J. Physiol. Endocrinol. Metab. 296, E936–E944 (2009). 19190262 10.1152/ajpendo.90768.2008

