# Supplementary material for: Sustained release of a GLP-1 and FGF21 dual agonist from an injectable depot protects mice from obesity and hyperglycemia
Source: Sci Adv. 2020 Aug 26;6(35):eaaz9890. doi: 10.1126/sciadv.aaz9890 (PMC7449677; doi:10.1126/sciadv.aaz9890)
Supplement: aaz9890_SM.pdf [file aaz9890_SM.pdf]

Science Advances
AAAS

advances.sciencemag.org/cgi/content/full/6/35/eaaz9890/DC1

Supplementary Materials for

Sustained release of a GLP-1 and FGF21 dual agonist from an injectable depot protects mice from obesity and hyperglycemia

C. A. Gilroy, M. E. Capozzi, A. K. Varanko, J. Tong, D. A. D'Alessio, J. E. Campbell, A. Chilkoti*

*Corresponding author. Email: chilkoti@duke.edu

Published 26 August 2020, *Sci. Adv.* **6**, eaaz9890 (2020)
DOI: 10.1126/sciadv.aaz9890

This PDF file includes:

Figs. S1 and S2
Tables S1 and S2

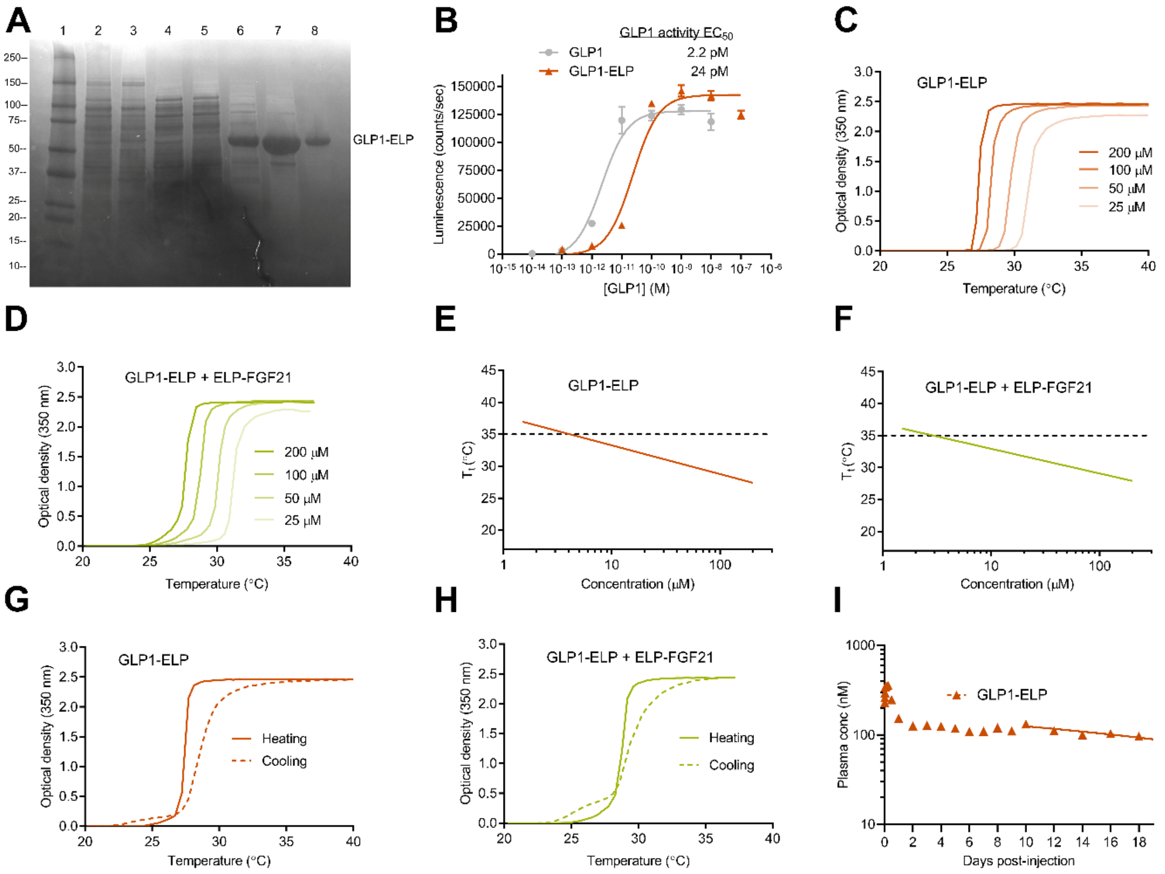

Fig. S1. Production and characterization of single agonist ELP fusion controls. (**A**) SDS-PAGE analysis of newly designed and synthesized GLP1-ELP. The 52 kDa fusion protein incorporated an ELP consisting of 120 VPGXG pentapeptide repeats with a 4:1 ratio of valine:alanine at the X_{aa} residue. GLP1-ELP was recombinantly produced and purified by ITC. 1: Molecular weight ladder (kDa). 2: Cell lysate. 3: Insoluble lysate fraction. 4: Soluble lysate fraction. 5-7: ITC rounds 1-3. (**B**) *In vitro* activity assay evaluating the EC_{50} of the newly synthesized GLP1-ELP fusion protein at the GLP-1R. GLP-1R agonism was measured by quantifying cAMP production following 5 h stimulation of HEK293 cells stably expressing the GLP-1R and a cAMP-inducible luciferase reporter. Data are presented as mean ± SEM, n=3. (**C-H**) LCST phase transition behavior of newly synthesized GLP1-ELP or a 1:1 mixture of newly synthesized GLP1-ELP and previously characterized ELP-FGF21(*18*). (**C-D**) The optical density at 350 nm was measured as a function of temperature. Dilutions were prepared in PBS and temperature was ramped at a rate of 1°C/min. The fusion mixture consists of each GLP1-ELP and ELP-FGF21 diluted to the indicated concentration. (**E-F**) Turbidity vs. temperature scans were repeated as in (**C-D**) for the indicated concentrations (n=3). T_ts were measured as the temperature corresponding to the 50% maximum optical density and plotted as a function of concentration. The horizontal dashed line indicates the approximate temperature of the s.c. space in a mouse(*27*). (**G-H**) Turbidity scans were repeated at an injection-relevant concentration (100-200 μM), ramping up to 37°C, then down to 20°C. (**I**) A GLP1-ELP fusion has a pharmacokinetic profile consistent with a sustained release depot. 6-week-old *db/db* mice (n=5) received a single s.c. injection of newly synthesized GLP1-ELP. The fusion was radiolabeled,

injected at 200 μM, and dosed at 1000 nmol/kg. Blood samples were collected at indicated time points following injection, and plasma gamma counts were measured and correlated to fusion protein concentration. Lines represent regression curves fit to the terminal portion of each data set. Data can be described by both a first-order (dotted) or a zero-order (solid) elimination model. Data are presented as mean ± SEM.

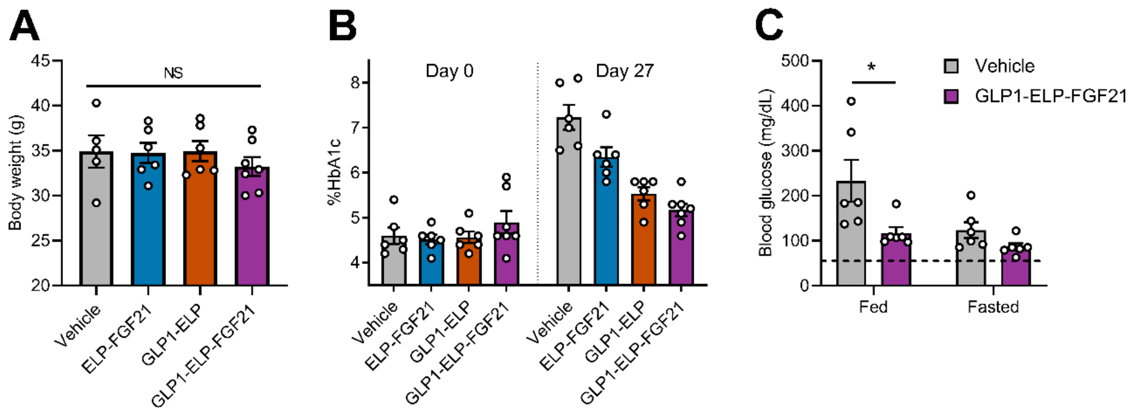

Fig. S2. Raw body weights, %HbA1c values, and fasting tolerance upon dual agonist treatment. (**A-B**) 6-week-old *db/db* mice (n=6-7) were treated weekly for 4 weeks with GLP1-ELP-FGF21, GLP1-ELP, ELP-FGF21, or PBS vehicle. Drugs were administered s.c. at 1000 nmol/kg. (**A**) Body weights are consistent between treatment groups at the GTT performed 3 days after the first injection (Day 3). (**B**) Chronic treatment with GLP1-ELP-FGF21 protects from %HbA1c elevation. %HbA1c was measured prior to the first treatment (Day 0) and 6 days following the final treatment (Day 27). (**C**) GLP1-ELP-FGF21 treatment does not predispose mice to nocturnal hypoglycemia. 8-week-old *db/db* mice (n=6) were injected s.c. with 1000 nmol/kg GLP1-ELP-FGF21 or vehicle, and *ad libitum*-fed blood glucose levels were measured 48 h following treatment administration ("Fed"). Mice were then subjected to an overnight 16 h fast, after which blood glucose measurements were repeated ("Fasted"). The horizontal dashed line indicates the conventionally defined threshold of hypoglycemia, 55 mg/dL(*33*). Data are presented as mean ± SEM and were analyzed by one-way ANOVA or two-way repeated measures ANOVA followed by Dunnett's tests. * = $p < 0.05$, NS = not significant.

Table S1. Amino acid sequences for ELP fusion proteins.

Fusion Protein	Amino Acid Sequence	Molecular Weight (kDa)
GLP1-ELP-FGF21	*AAHGEGTFTSDVSSYLEEQAAKEFIAWLVKGA*GVGVPGV<br>GVPGAGVPGVGVPGVGVPGVGVPGVGVPGAGVPGV<br>GVPGVGVPGVGVPGVGVPGAGVPGVGVPGVGVPGV<br>GVPGVGVPGAGVPGVGVPGVGVPGVGVPGVGVPGA<br>GVPGVGVPGVGVPGVGVPGVGVPGAGVPGVGVPGV<br>GVPGVGVPGVGVPGAGVPGVGVPGVGVPGVGVPGV<br>GVPGAGVPGVGVPGVGVPGVGVPGVGVPGAGVPGV<br>GVPGVGVPGVGVPGVGVPGAGVPGVGVPGVGVPGV<br>GVPGVGVPGAGVPGVGVPGVGVPGVGVPGVGVPGA<br>GVPGVGVPGVGVPGVGVPGVGVPGAGVPGVGVPGV<br>GVPGVGVPGVGVPGAGVPGVGVPGVGVPGVGVPGV<br>GVPGAGVPGVGVPGVGVPGVGVPGVGVPGAGVPGV<br>GVPGVGVPGVGVPGVGVPGAGVPGVGVPGVGVPGV<br>GVPGVGVPGAGVPGVGVPGVGVPGVGVPGVGVPGA<br>GVPGVGVPGVGVPGVGVPGVGVPGAGVPGVGVPGV<br>GVPGVGVPGVGVPGAGVPGVGVPGVGVPGVGVPGV<br>GVPGAGVPGVGVPGVGVPGVGVPGVGVPGAGVPGV<br>GVPGVGVPGVGVPGVGVPGAGVPGVGVPGVGVPG**A**<br>**YPIPDSSPLLQFGGQVRQRYLYTDDDQDTEAHLEIR**<br>**EDGTVVGAAHRSPESLLELKALKPGVIQILGVKASR**<br>**FLCQQPDGALYGSPHFDPEACSFRERLLEDGYNVY**<br>**QSEAHGLPLRLPQKDSPNQDATSWGPVRFLPMPGL**<br>**LHEPQDQAGFLPPEPPDVGSSDPLSMVEGSQGRSPS**<br>**YASG**	71.9
ELP-FGF21	GVGVPGVGVPGAGVPGVGVPGVGVPGVGVPGVGVP<br>GAGVPGVGVPGVGVPGVGVPGVGVPGAGVPGVGVP<br>GVGVPGVGVPGVGVPGAGVPGVGVPGVGVPGVGVP<br>GVGVPGAGVPGVGVPGVGVPGVGVPGVGVPGAGVP<br>GVGVPGVGVPGVGVPGVGVPGAGVPGVGVPGVGVP<br>GVGVPGVGVPGAGVPGVGVPGVGVPGVGVPGVGVP<br>GAGVPGVGVPGVGVPGVGVPGVGVPGAGVPGVGVP<br>GVGVPGVGVPGVGVPGAGVPGVGVPGVGVPGVGVP<br>GVGVPGAGVPGVGVPGVGVPGVGVPGVGVPGAGVP<br>GVGVPGVGVPGVGVPGVGVPGAGVPGVGVPGVGVP<br>GVGVPGVGVPGAGVPGVGVPGVGVPGVGVPGVGVP<br>GAGVPGVGVPGVGVPGVGVPGVGVPGAGVPGVGVP<br>GVGVPGVGVPGVGVPGAGVPGVGVPGVGVPGVGVP<br>GVGVPGAGVPGVGVPGVGVPGVGVPGVGVPGAGVP<br>GVGVPGVGVPGVGVPGVGVPGAGVPGVGVPGVGVP<br>GVGVPGVGVPGAGVPGVGVPGVGVPGVGVPGVGVP<br>GAGVPGVGVPGVGVPGVGVPGVGVPGAGVPGVGVP<br>GVGVPG**AYPIPDSSPLLQFGGQVRQRYLYTDDDQD**<br>**TEAHLEIREDGTVVGAAHRSPESLLELKALKPGVIQ**	68.5

	**ILGVKASRFLCQQPDGALYGSPHFDPEACSFRERLL EDGYNVYQSEAHGLPLRLPQKDSPNQDATSWGPV RFLPMPGLLHEPQDQAGFLPPEPPDVGSSDPLSMV EGSQGRSPSYASG**	
GLP1-ELP	*AAHGEGTFTSDVSSYLEEQAAKEFIAWLVKGA*GVGVPGV GVPGAGVPGVGVPGVGVPGVGVPGVGVPGAGVPGV GVPGVGVPGVGVPGVGVPGAGVPGVGVPGVGVPGV GVPGVGVPGAGVPGVGVPGVGVPGVGVPGVGVPGA GVPGVGVPGVGVPGVGVPGVGVPGAGVPGVGVPGV GVPGVGVPGVGVPGAGVPGVGVPGVGVPGVGVPGV GVPGAGVPGVGVPGVGVPGVGVPGVGVPGAGVPGV GVPGVGVPGVGVPGVGVPGAGVPGVGVPGVGVPGV GVPGVGVPGAGVPGVGVPGVGVPGVGVPGVGVPGA GVPGVGVPGVGVPGVGVPGVGVPGAGVPGVGVPGV GVPGVGVPGVGVPGAGVPGVGVPGVGVPGVGVPGV GVPGAGVPGVGVPGVGVPGVGVPGVGVPGAGVPGV GVPGVGVPGVGVPGVGVPGAGVPGVGVPGVGVPGV GVPGVGVPGAGVPGVGVPGVGVPGVGVPGVGVPGA GVPGVGVPGVGVPGVGVPGVGVPGAGVPGVGVPGV GVPGVGVPGVGVPGAGVPGVGVPGVGVPGVGVPGV GVPGAGVPGVGVPGVGVPGVGVPGVGVPGAGVPGV GVPGVGVPGVGVPGVGVPGAGVPGVGVPGVGVPG	51.9

Underlined, italicized, and bold designates residues associated with ELP, GLP-1, and FGF21, respectively.

Table S2. Pharmacokinetic regression fits and parameters for ELP fusion protein depots following s.c. administration to mice.

Labeled construct	*Regression model*	*R-squared*	*C_{max} (nM)*	*t_{max} (h)*	*AUC (nM*d)*	*$t_{1/2, abs}$ (d)*	*Regression model*	*R-squared*
GLP1-ELP-FGF21	Log(y) = -0.04160x + 2.431	0.85	411 ± 51	10.5 ± 1.5	1961 ± 59	7.6 ± 1.1	y = -7.273x + 174.1	0.81
GLP1-ELP	Log(y) = -0.01628x + 2.263	0.61	369 ± 14	4.8 ± 0.7	2023 ± 67	20.8 ± 3.2	y = -3.932x + 164.1	0.58
GLP1-ELP as part of 1:1 single agonist fusion mixture	Log(y) = -0.01803x + 2.013	0.87	233 ± 13	21 ± 9	1552 ± 66	17.0 ± 1.4	y = -2.584x + 93.91	0.86
ELP-FGF21 as part of 1:1 single agonist fusion mixture	Log(y) = -0.02515x + 1.671	0.63	338 ± 23	12 ± 0	1135 ± 59	13.2 ± 2.7	y = -1.264x + 38.80	0.60

Data are reported as means ± SEM. C_{max}, observed maximum serum concentration; t_{max}, time to C_{max}; AUC, area under the curve; $t_{1/2, abs}$, absorption half-life.
